# Synthetic antibodies targeting EphA2 induce diverse signaling‐competent clusters with differential activation

**DOI:** 10.1002/pro.70145

**Published:** 2025-05-24

**Authors:** Jarrett J. Adams, Heather A. Bruce, Suryasree Subramania, Lynda Ploder, Julia Garcia, Isabelle Pot, Levi L. Blazer, Alexander U. Singer, Sachdev S. Sidhu

**Affiliations:** ^1^ School of Pharmacy University of Waterloo Kitchener Ontario Canada

**Keywords:** antibody, Efn, Eph, EphA2, mechanism, phage display, receptor clustering, receptor tyrosine kinase, signaling

## Abstract

The receptor tyrosine kinase EphA2 interacts with ephrin (Efn) ligands to mediate bi‐directional signals that drive cellular sorting processes during tissue development. In the context of various cancers, EphA2 can also drive invasive metastatic disease and represents an important target for cancer therapeutics. Natural Efn ligands sterically seed intertwined EphA2 clusters capable of recruiting intracellular kinases to mediate trans‐phosphorylation. Synthetic proteins, such as antibodies (Abs), can mimic Efn ligands to trigger EphA2 signaling, leading to receptor internalization and degradation, and enabling intracellular delivery of conjugated drugs. Furthermore, Abs are capable of recruiting EphA2 into clusters distinct from those seeded by Efn. We developed three synthetic Abs targeting distinct EphA2 domains and determined the paratope valency necessary for agonist or antagonist properties of each of the three epitopes. Structural modeling of monovalent Fabs in complex with EphA2 elucidated competitive and non‐competitive mechanisms of inhibition of EphA2 canonical signaling. Likewise, modeling of clusters induced by bivalent IgGs elucidated multiple signaling‐competent EphA2 clusters capable of triggering a continuum of signaling strengths and provided insights into the requirement for multimerization of EphA2 to trigger phosphorylation. Our study shows how different agonist clusters lead to distinct kinase recruitment efficiencies to modify phosphotyrosine signal strength, and provides a panel of anti‐EphA2 Abs as reagents for the development of therapeutics.

## 1 INTRODUCTION

Erythropoietin‐producing hepatocellular (Eph) receptors and their ephrin (Efn) ligands are modulators of cellular segregation and tissue patterning. The 14 Eph receptors share a common architecture and form the largest subfamily of human receptor tyrosine kinases (RTKs). Unlike most RTKs that bind soluble growth factors, Eph receptors primarily bind Efn ligands anchored to the cell membrane either by a glycosylphosphatidylinositol (GPI) link (EfnA type) or by a single‐pass transmembrane (TM) domain (EfnB type) (Kullander & Klein, 2002). Cellular Eph‐Efn engagement initiates signaling through both “forward” Eph phosphotyrosine (pTyr) signaling and “reverse” Efn signaling to bi‐directionally orchestrate cellular adhesion and repulsion responses (reviewed in Jorgensen et al., 2009; Pasquale, 2008). Though extensively studied, a mechanistic understanding of the diverse cellular responses produced by Eph‐Efn signaling has yet to be fully realized, owing to the complexity of the signaling complexes and the large number of receptors and ligands involved. Nonetheless, several Eph receptors — notably EphA2 — have been proposed as therapeutic targets in oncology due to their aberrant expression in many cancers, which has been correlated with invasive and metastatic disease (Liu et al., 2022; Pergaris et al., 2021).

The most advanced therapeutic strategy using EphA2 targeting has been for the delivery of antibody‐drug conjugates (ADCs). Antibodies (Abs) that agonize and trigger internalization of EphA2 can be conjugated to cytotoxic drugs, and these ADCs have been used to deliver the drugs to cancer cells overexpressing EphA2 (Jackson et al., 2008; Lee et al., 2009). While the preclinical efficacy of an ADC derived from an agonist EphA2 Ab (1C1) appeared promising, adverse events observed in a phase I clinical trial halted its development as a drug (Annunziata et al., 2013). More recently, EphA2‐targeted delivery of a cytotoxic drug using a bicyclic peptide has been applied safely in a phase I clinical trial (Bennett et al., 2020; Dolgin, 2021). Moreover, another Ab (DS‐8895a) that acts as a partial antagonist of EphA2 has been used safely in the clinic (Hasegawa et al., 2016; Shitara et al., 2019) but has not progressed beyond a phase I trial. Thus, EphA2 remains a promising but under‐utilized target for Ab‐based cancer therapeutics, and we reasoned that the development of a more extensive toolkit of Abs with detailed structural knowledge could provide a better understanding of EphA2 signaling and potentially could enable the development of more efficacious therapeutics with better safety profiles.

**FIGURE 1:**
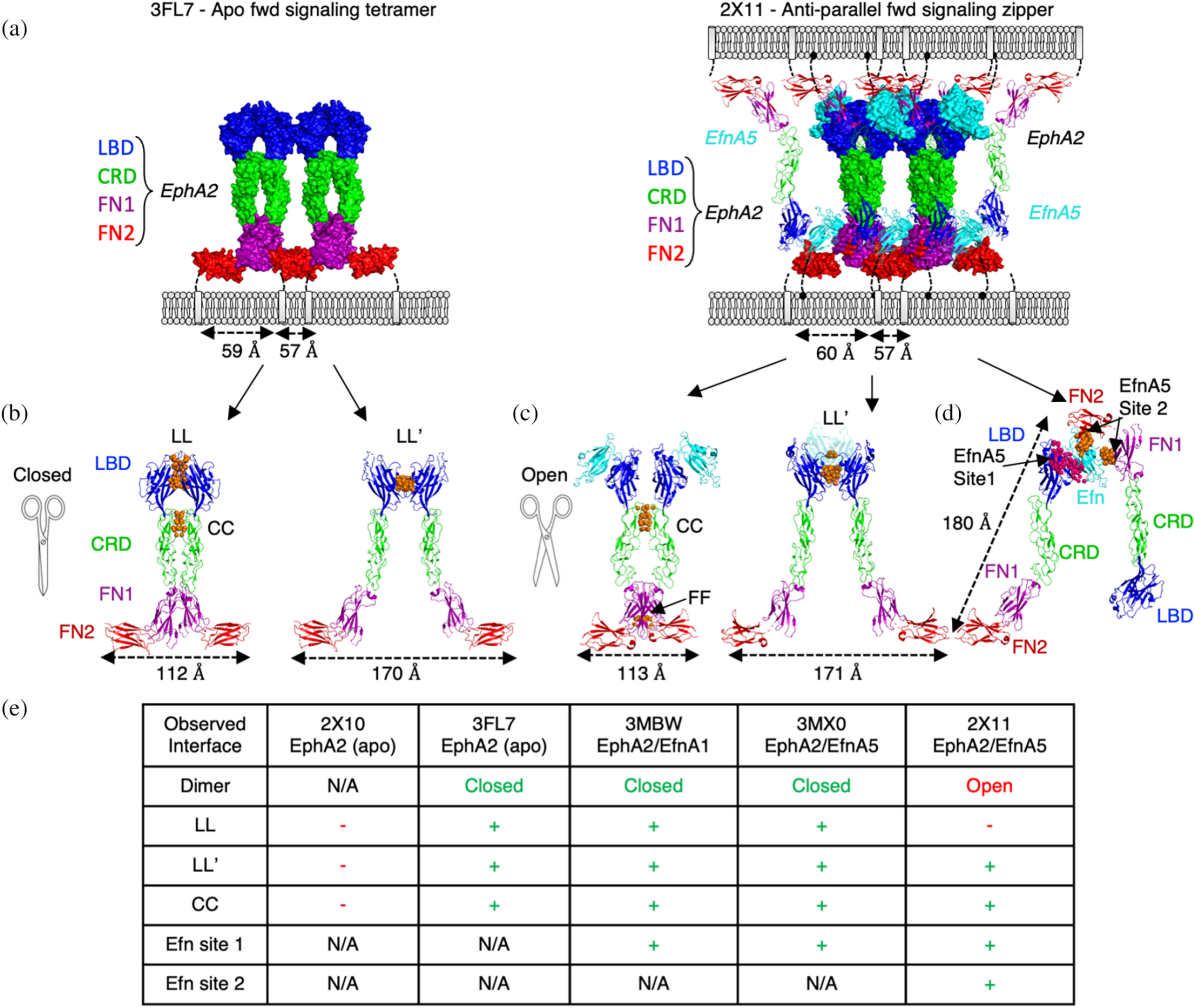
Interfaces in EphA2 clusters. (a) Surface rendering of EphA2 clusters derived from the apo EphA2 extracellular domain (ECD) complex (protein data bank (PDB) entry 3FL7, *left*) and the EphA2‐EfnA5 complex (PDB entry 2X11, *right*). Domains of EphA2 (ligand‐binding domain (LBD), blue; cysteine‐rich domain (CRD), green; N‐terminal fibronectin‐3 domain (FN1), purple; C‐terminal fibronectin‐3 domain (FN2), red) and EfnA5 (cyan) are illustrated as tetrameric chain‐link clusters. For the 2X11 cluster, trans‐engaging receptors and cis‐engaging EfnA5 are illustrated as ribbons without surfaces. Distances between C‐termini approximating transmembrane (TM) distance of clustered receptors within the tetramer are indicated (dashed arrows). (b) Ribbon rendering of the “closed” dimer mediated by LL and CC interfaces (*left*) or LL′ interfaces (*right*) with interface contacts atoms (<4.5 Å) between EphA2 protomers shown as orange spheres. Distances between C‐termini approximating TM distance within the dimer are indicated (dashed arrows). (c) Ribbon rendering of the “open” dimer through CC/FN1 (*left*) or LL′ interfaces (*right*) with interface contact residues (<4.5 Å) between EphA2 protomers shown as orange spheres. Distances between C‐termini approximating TM distance within the dimer are indicated (dashed arrows). (d) Ribbon rendering of trans‐zippered EphA2. EfnA5 associates with high affinity to the LBD (Site 1) and forms anti‐parallel interactions with adjacent protomers of EphA2 through the FN1 and FN2 domains (Site 2). Interface contact residues (<4.5 Å) between EphA2 and EfnA5 protomers are shown as pink spheres for Site 1 and orange spheres for Site 2. Distances between C‐termini approximating distance within the head‐to‐tail dimer are indicated (dashed arrows). (e) Summary table of observed interfaces for each crystal structure of EphA2 in either apo or ephrin (Efn) bound forms. Interfaces between adjacent protomers made through the LBD (LL and LL′), CRD (CC) or to Efn (Site 1 and Site 2) are annotated as observed (+) or absent (−) in each unit cell (PDB entries 2X10, 3FL7, 3MBW, 3MX0, and 2X11). Structures of EphA2 where dimer interfaces are not possible are denoted as not applicable (N/A).

Extensive structural studies of Eph receptors and Eph‐Efn complexes have provided important insights into how signaling is triggered in different cellular contexts (reviewed in Kania & Klein, 2016). The Eph extracellular domain (ECD) consists of four subdomains: the ligand‐binding domain (LBD), the cysteine‐rich domain (CRD) that includes a sushi domain and an epidermal‐growth‐factor (EGF)‐like domain (Arora et al., 2023), the N‐terminal fibronectin‐3 domain (FN1), and the C‐terminal fibronectin‐3 domain (FN2) (Figures 1a and S1A). Efn ligands bind to the LBD and sterically seed clusters of Eph ECDs, which then drive forward signaling through tyrosine autophosphorylation of the Eph intracellular domains (ICDs). Alternatively, cells presenting both Eph and Efn can form adhesion zipper clusters to initiate anti‐parallel Eph forward signaling (Figures 1a–d and S1B). Parallel forward signaling and anti‐parallel forward signaling can be structurally defined by two models of EphA2 active signaling states that we categorize as “closed” or “open,” and which drive chain‐linked clusters or zippered chain‐linked clusters, respectively (Figure 1a–d). Overexpression of EphA2 can lead to the spontaneous formation of clusters capable of pTyr signaling in the absence of Efn stimulation (Wimmer‐Kleikamp et al., 2004). A closed model of the active but apo EphA2 cluster was characterized by crystallizing the full‐length ECD of EphA2 (protein data bank (PDB) entry 3FL7) (Figure 1a left panel; 1b). In this lattice, the EphA2 ECD formed an array through protomer‐protomer associations at three distinct interfaces. Two protomers formed a symmetric homodimer by utilizing two interfaces, one between the LBDs (LL interface) and a second between the CRDs (CC interface). Each dimer also interacted with adjacent dimers through another tandem interface between the LBDs (LL′ interface), creating a “chain‐link” array of parallel receptors. Active EphA2 signaling clusters therefore have multiple LBD interfaces that can be induced by overexpression or by Efn engagement.

Ligand‐mediated dimerization is a common mechanism of RTK signaling, where ligand engagement stabilizes the association of two receptors, which in turn drives intracellular signaling through induced proximity of the kinase domains. Interestingly, Efns are not obligate homodimers, and they do not induce large structural changes in the receptor ECD to enable its dimerization. Instead, they interact almost exclusively with a single EphA2 protomer within the presumed parallel forward signaling tetramer and only modestly cross‐link adjacent EphA2 protomers through a single loop (Figure S1C). Efns also utilize carbohydrate contacts to cross‐link EphA2 and stabilize LL′‐mediated dimers (Figure S1C) (Ferluga et al., 2013). However, Efn‐mediated dimers of EphA2 do not recruit the TM domains into a proximity compatible with signaling, but rather, close proximity is achieved by tetrameric association of a dimer of dimers (Figures 1a–e and S1B). We therefore hypothesize that tetrameric clusters of EphA2, not dimers, are the minimal signaling‐competent arrangement induced by Efn, as previously postulated for other Eph isoforms (Schaupp et al., 2014).

Importantly, the protein–protein interfaces that recruit EphA2s into higher order clusters and signaling units are largely independent of Efn (Himanen et al., 2010; Wimmer‐Kleikamp et al., 2004). Efn ligands sterically seed clusters by stabilizing the LL′ interface to drive dimerization but tetramerization and higher order clustering relies on EphA2 self‐association through tandem LL/CC and LL′ interfaces. Notably, the partial EphA2 ECD complexes bound to EfnA1 (PDB entry 3MX0) or EfnA5 (PDB entry 3MBW) present the same closed tetrameric structure in their lattices when symmetry related molecules are considered, demonstrating a clear structural convergence of the closed chain‐linked tetramer as the minimal forward signaling complex (Figure S1B) (Himanen et al., 2010).

Intriguingly, EphA2 forms another type of forward signaling‐competent cluster. The open model for EphA2 signaling is unique in its interactions but similar to the closed cluster in its receptor geometric placement (Figure 1b). This cluster could arise in anti‐parallel adhesion clusters that “zipper” cis‐expressed EfnA5 and EphA2 into trans synapses between matched cells (Seiradake et al., 2010). In this structure, the ECDs of EphA2 engage EfnA5 (PDB entry 2X11, Figures 1a right, c,d, and S1B) to array in a dimer‐of‐dimer arrangement distinct from that of the closed tetramer. This alternative open tetramer is stabilized by the LL′ interface as observed in the closed tetramer (Figure 1b), but it differs in that the LL interface does not occur (Figure 1e). Instead, the open tetrameric complex is stabilized by two interfaces within the dimer involving the CRDs (CC interface) and, to a lesser extent, the FN1s (Figure 1c,e) (Seiradake et al., 2010). The CC interfaces are not identical in the open and closed clusters but largely overlap and act as a molecular fulcrum, allowing protomers within a dimer to scissor open or closed to satisfy distinct cluster geometries induced by specific Efn conditions.

Though lacking the LL interface, the open EphA2‐EfnA5 cluster bridges two parallel EphA2 protomers through a high affinity Site 1 interaction and it also bridges two anti‐parallel EphA2 protomers through a low affinity Site 2 interaction (Figure 1d,e). The strength of the Site 2 Efn interaction and subsequent zippering has not been determined, but it is presumed to be very weak, and perhaps, only detectable when Eph and Efn are present on the same cellular membrane and/or under the extremely avid conditions of a cellular synapse. Importantly, mutations in the Site 2 interface only modestly reduce EphA2 clustering and pTyr signaling (Seiradake et al., 2010). Therefore, under the conditions of an *in vitro* cellular stimulation assay with recombinant EfnA5‐Fc, the bulk of EfnA5 forward pTyr signaling is induced through Site 1 engagement and subsequent parallel closed tetramer clustering, but a distinct active state is achieved if both parallel (closed tetramer assembly) and anti‐parallel (open tetramer assembly) cluster configurations are possible (Seiradake et al., 2010).

Despite the structural evidence for our model of tetrameric signaling complexes, functional studies of Eph receptor signaling have generally adopted an aggregating dimer model of activation, where dimerization is necessary and adequate for minimal triggering of pTyr activity but is amplified by further propagation of the cluster with either liganded or unliganded Eph receptors to form higher ordered clusters (Day et al., 2005; Nikolov et al., 2013; Seiradake et al., 2010). In addition, some alternative higher‐order clusters may also serve to negatively regulate signaling through cis head‐to‐tail protomer‐protomer associations for at least some Eph isoforms (Nikolov et al., 2013; Ojosnegros et al., 2017). Clusters of EphA2 clusters can therefore be either stimulatory or inhibitory to kinase activation, depending on the interfaces involved and the geometric configurations they take.

Cellular and biophysical studies of EphA2 signaling in the context of a membrane have revealed a range of EphA2 cluster sizes, from monomers to hundred‐protomer assemblies (Nikolov et al., 2014), with cluster size correlated to signal strength (Zapata‐Mercado et al., 2022). As expected, clustering and signaling induced by Efn and alternative agonists are modulated by mutations in the LL, LL′, and CC interfaces (Gomez‐Soler et al., 2019; Seiradake et al., 2010; Singh et al., 2018). However, divergent agonist ligands are differentially dependent on these interfaces, implying some plasticity in the composition of signaling‐competent clusters (Gomez‐Soler et al., 2022). This diversity of functional observations has yet to be reconciled with the dimeric or tetrameric structural models of activation, creating some residual mechanistic ambiguity.

Inhibitor Abs of EphA2 have also been developed to target the LBD and compete directly with Efn ligands (Ab D2) (Goldgur et al., 2014) or, in the case of Ab DS‐8895a (Hasegawa et al., 2016), to target the membrane‐proximal FN2 domain and, presumably, sterically occlude the kinase proximity necessary for phosphorylation; but neither has successfully translated into approved therapies, nor have their mechanistic actions on EphA2 dimerization or tetramerization been reconciled. We sought to systematically characterize monovalent and bivalent Ab modalities that promote or interfere with receptor clustering by specifically targeting each of the domains of the EphA2 ECD in order to fully exploit EphA2 as a therapeutic target and clarify the triggering mechanism of this intriguing RTK.

We have developed optimized phage display technologies that allow for the rapid development of precisely engineered synthetic Abs to drive receptor clustering through distinct epitopes (Miersch et al., 2017, 2022). Moreover, we have engineered antigen‐binding fragment (Fab) frameworks as optimized chaperones for enhanced crystal packing to enable rapid and efficient elucidation of Fab:antigen crystal structures (Bruce et al., 2023, 2024). To elucidate the details of the mechanisms of Eph receptor clustering and activation, we developed a panel of synthetic Abs targeting the ECD subdomains of EphA2, and produced monovalent Fabs and bivalent IgGs to either negatively or positively modulate signaling. Using the Fab crystal chaperone technology, we crystallized Fab:EphA2 complexes and evaluated the molecular determinants of both agonists and antagonists of EphA2.

## 2 RESULTS

### 2.1 Affinity and specificity of anti‐EphA2 Abs

**FIGURE 2:**
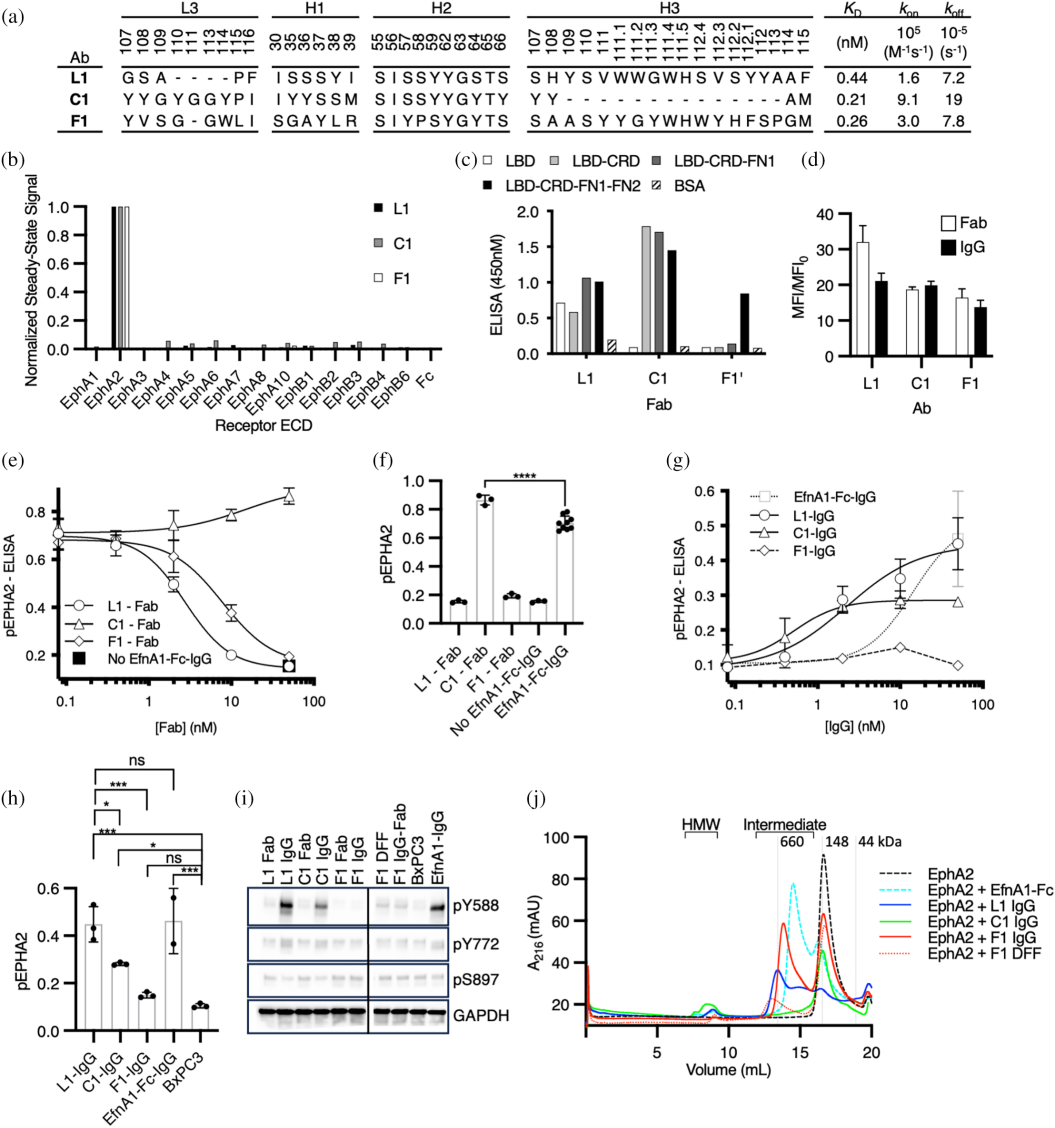
Functional characterization of anti‐EphA2 antibodies (Abs). (a) Complementarity‐determining region (CDR) sequences of anti‐EphA2 antigen‐binding fragments (Fabs). Sequences are shown for positions that were diversified in the phage library and are numbered according to the international ImMunoGeneTics (IMGT) information system nomenclature (Lefranc, 2014). Kinetic binding constants for Fabs binding to immobilized EphA2 extracellular domain (ECD) were determined by surface plasmon resonance and are shown on the right. (b) Specificity of IgGs assessed with a panel of ECDs from the 14 human erythropoietin‐producing hepatocellular (Eph) receptor family members. Steady‐state biolayer interferometry was used with each immobilized Eph ECD (*x*‐axis) to measure the binding of 100 nM IgG, and binding signal was normalized to the signal for immobilized EphA2 and Fc (*y*‐axis). (c) Specificity of 100 nM Fabs assessed by enzyme‐linked immunosorbent assay (ELISA) with immobilized domain fragments of EphA2. Fab F1′ refers to the parental Fab raised from Library F before optimization (Enderle et al., 2021). (d) Flow cytometry of BxPC3 tumor cells treated with 100 nM Ab L1, C1 or F1 in the Fab (white) or IgG format (black). The fold median fluorescence signal relative to secondary alone is plotted (*n* = 2). (e) Effects of Fabs (*x*‐axis) on phosphorylated EphA2 (pEphA2) levels in BxPC3 cells stimulated with EfnA1‐Fc‐IgG, assessed by quantitative ELISA detection of EphA2 pTyr^588^ (*y*‐axis). (f) Maximum inhibition levels of pEphA2 plotted as a bar graph with individual replicates illustrated by dots for antagonistic Fabs. Statistical analysis was carried out using analysis of variance (ANOVA) (*n* ≥ 3) in Prism GraphPad where **** indicates *p* < 0.0001. (g) Effects of IgGs (*x*‐axis) on pEphA2 levels in BxPC3 cells, assessed by quantitative ELISA detection of EphA2 pTyr^588^ (*y*‐axis). Activation was fit using linear regression (solid line) or trend line if “hooked” (dashed) due to saturation‐dependent loss of activity. (h) Statistical evaluation of maximum activation levels of pEphA2 plotted as a bar graph with individual replicates illustrated by dots for agonist IgGs (left). Statistical analysis was carried out using ANOVA (*n* ≥ 3) in Prism GraphPad. Significant differences in maximal agonist activation were observed for IgGs L1 and C1 (ANOVA, *p* < 0.05). Statistical analysis was carried out by ANOVA where ns (no significance) indicates *p* > 0.05, * indicates *p* < 0.05, and *** indicates *p* < 0.001. (i) Phospho‐specific western analysis of pEphA2 in response to saturating concentrations of Ab agonists (100 nM) in BxPC3 cells. Blots were probed for juxtamembrane‐pTyr^588^, kinase‐pTyr^772^ and kinase‐pS^897^signal in response to 15 min of ligand stimuli in conditioned media. (j) Size exclusion chromatograpy of clusters induced by EphA2 IgGs (200 nM) or diabody‐Fc‐Fab F1 (100 nM) and EfnA1‐Fc (200 nM) bound to EphA2 (500 nM) separated on Superose 6 10/300 column. Peak volumes of standard molecular weight proteins are indicated (gray). Intermediate complexes ≤660 kDa and high molecular weight complexes are indicated. CRD, cysteine‐rich domain; LBD, ligand‐binding domain.

To create Abs to manipulate EphA2 clustering, we carried out selections to target each of the three major interface domains of the EphA2 ECD (LBD, CRD, and FN2) using established methods with highly functional synthetic Fab‐phage libraries (Adams et al., 2014; Enderle et al., 2021; Himanen et al., 2010), resulting in dozens of unique Fabs targeting each domain. To assess specificity comprehensively, we converted selected Fabs to the full‐length IgG format, purified IgG proteins, and screened numerous IgGs for binding to the complete panel of Eph receptor ECDs at high concentration (100 nM) and at steady state using biolayer interferometry (BLI). Ultimately, we focused on three distinct Abs (Figure 2a) that bound to the EphA2 ECD with high affinity but not to any of the other Eph family members (Figures 2b and S2A), and were capable of modulating EphA2 pTyr signaling. We assessed the binding of the three IgGs to purified domains of the EphA2 ECD and showed that one bound to the LBD (L1), another bound to the CRD (C1), and the third bound to FN2 (F1) (Figures 2c and S2B,C). Finally, to determine monovalent binding kinetics, we purified each Fab protein and assayed binding to the EphA2 ECD by surface plasmon resonance (SPR) (Figures 2a and S2A). Each of the Fabs bound to the EphA2 ECD with high affinity (*K*_D_ <1 nM), and together, they comprised a toolkit for manipulating EphA2 signaling.

### 2.2 Cellular activity of anti‐EphA2 Abs

Next, we characterized the Abs in cell‐based assays to identify bivalent IgG agonists and monovalent Fab antagonists. In addition to homodimerization, EphA2 can heterodimerize with other Eph receptors with potentially distinct ligand specificities and cellular responses (Janes et al., 2011). To avoid complications associated with signaling involving heterodimers and/or auto‐activation arising from overexpression, we sought to characterize our Abs with cells that displayed uniform but endogenous EphA2. To identify a suitable cell line, we profiled a panel of pancreatic tumor cell lines with a set of 14 Abs we had developed, each selective for one of the 14 Eph family members (Figure S2D–F). We found that BxPC3 cells almost exclusively displayed EphA2, whereas all other cell lines displayed a range of Eph family members (Figure S2E). We then confirmed that each of our anti‐EphA2 Abs, in either the Fab or IgG format, bound robustly to BxPC3 cells (Figure 2d).

Next we used a semi‐quantitative enzyme‐linked immunosorbent assay (ELISA) to assess and quantify the ability of monovalent anti‐EphA2 Fabs to antagonize EfnA1‐mediated activation of EphA2 in BxPC3 cells. We measured EphA2 pTyr^588^ levels in the presence of the multivalent agonist EfnA1‐Fc clustered with anti‐Fc IgG (EfnA1‐Fc‐IgG) and a concentration range of each Fab (Figure 2e,f). We found that Fabs L1 and F1 were potent and complete antagonists of EphA2 phosphorylation (EC_50_ = 2.8 and 7.8 nM, respectively), whereas Fab C1 did not act as an antagonist, but rather, appeared to modestly enhance EphA2 phosphorylation. Likewise, we quantified the agonist activity of bivalent EphA2 IgGs. BxPC3 cells were treated with a concentration range of bivalent anti‐EphA2 IgG or the positive control EfnA1‐Fc‐IgG, and EphA2 phosphorylation was measured using the pTyr^588^ ELISA (Figure 2g,h). IgG L1 resulted in potent (EC_50_ = 2.2 nM) phosphorylation of EphA2 and IgG C1 induced potent (EC_50_ = 0.5 nM) but partial phosphorylation of EphA2, whereas IgG F1 only weakly stimulated phosphorylation of EphA2 and the signal decayed upon saturation, as commonly observed for bivalent agonists due to interference. Notably, IgG C1 signal amplitude was approximately half that of IgG L1 at saturation but neither stimulus was significantly different from EfnA1‐Fc clustered with secondary IgG. For comparison, we evaluated the inhibitory properties of IgG F1 to determine if it would act similarly to an inverse agonist. Indeed, the combined stimulation of IgG F1 and EfnA1‐Fc‐IgG resulted in a partial inactivation of signaling (Figure S2G). We therefore characterized three distinct EphA2‐IgG complexes with diverse functional outcomes. To ensure that the ranking of EphA2 agonists was reproducible in cells expressing a mixture of Eph isoforms (Figure S2E), we repeated the maximal stimulations of pTyr^588^ on both BxPC3 (EphA2^+^) and MDA MB231 (EphA2^+^ and EphB2^+^) and quantified the activation by phospho‐specific western (Figures 2i, S2H,I). The trends in maximal stimulation were conserved across both cell lines. To further evaluate the signaling response to IgG stimuli at alternative sites of modification, we assessed the pEphA2 response at pTyr^772^ and pSer^897^ (Figures 2i and S2H,I). Activation through L1 and C1 IgG engagement mimicked stimulation by EfnA1‐Fc, suggesting that they trigger a similar intracellular signal. Activation through F1 engagement appeared to selectively stimulate pTyr^588^, albeit weakly.

To better understand the impact of agonist engagement on EphA2 clustering, we used size exclusion chromatography to observe the agonized IgG‐EphA2 complexes in solution. Agonist IgG was saturated with EphA2 ECD, allowing the complexes to multimerize before the distinct protomer states were separated by size over the column (Figure 2g). Analysis of the EphA2 IgGs and EfnA1‐Fc seeded complexes revealed multiple protomer states. For complexes induced by EfnA1‐Fc and IgG L1, prominent intermediate clusters of less than or equal to 660 kDa were observed, as well as a smaller proportion of high molecular weight (HMW) species consistent with induced EphA2 multimers. In contrast, IgG C1 gave rise to a prominent HMW species peak and relatively few intermediate products compared to IgG L1 and EfnA1‐Fc. Finally, IgG F1 induced almost exclusively intermediate sized complexes larger than those seeded by EfnA1‐Fc, but smaller than the ones induced by IgG L1. Furthermore, IgG F1 produced very few HMW products compared to other agonist complexes. Taken together, these data suggest that the HMW species might be responsible for phosphorylation of EphA2. Given that IgG F1 failed to produce a strong agonist signal or a prominent HMW peak, we asked if increased valency might potentiate agonistic properties through FN2 engagement. As anticipated, tetravalent modalities of F1 in a diabody‐Fc‐Fab format (DFF) produced a robust EphA2 phosphorylation signal in BxPC3 cells, albeit suboptimal compared to EfnA1‐Fc‐IgG, L1 IgG, or C1 IgG (Figure S2H,J,K). We characterized the chromatography profile of DFF F1 in complex with EphA2 ECD to evaluate the proportional distribution of induced EphA2 clusters. We observed a decrease in intermediate molecular weight products and a detectable increase in HMW products compared to IgG F1, suggesting that enhanced multimers of EphA2 were induced by DFF F1 engagement, resulting in enhanced phosphorylation. Together, these observations point to a relationship between the HMW clusters of EphA2 induced by agonist ligands, the valency of the agonists, and the maximal activation observed on cells.

### 2.3 Structural analysis of Fabs in complex with EphA2 domains

To shed light on the molecular basis for the diverse cellular activities of the various Abs, we solved the structure of each Fab in complex with its cognate EphA2 domain. To facilitate crystallization, we took advantage of optimized Fab frameworks that enhance the quantity and quality of Fab‐antigen crystals (Bruce et al., 2023, 2024). Briefly, we incorporated substitutions into the elbow region of the Fab heavy chain to reduce conformational flexibility (Bailey et al., 2018), in addition to making substitutions in the light chain constant domain that reduce the surface entropy and induce the formation of β‐sheet stacking interactions between packing Fab molecules in the crystal lattice (Bruce et al., 2023; Lieu et al., 2020). As these regions are distal to the Fab paratope, they do not affect antigen recognition in solution but improve the yield of Fab‐antigen crystals from conventional screens (Bruce et al., 2023, 2024).

**FIGURE 3:**
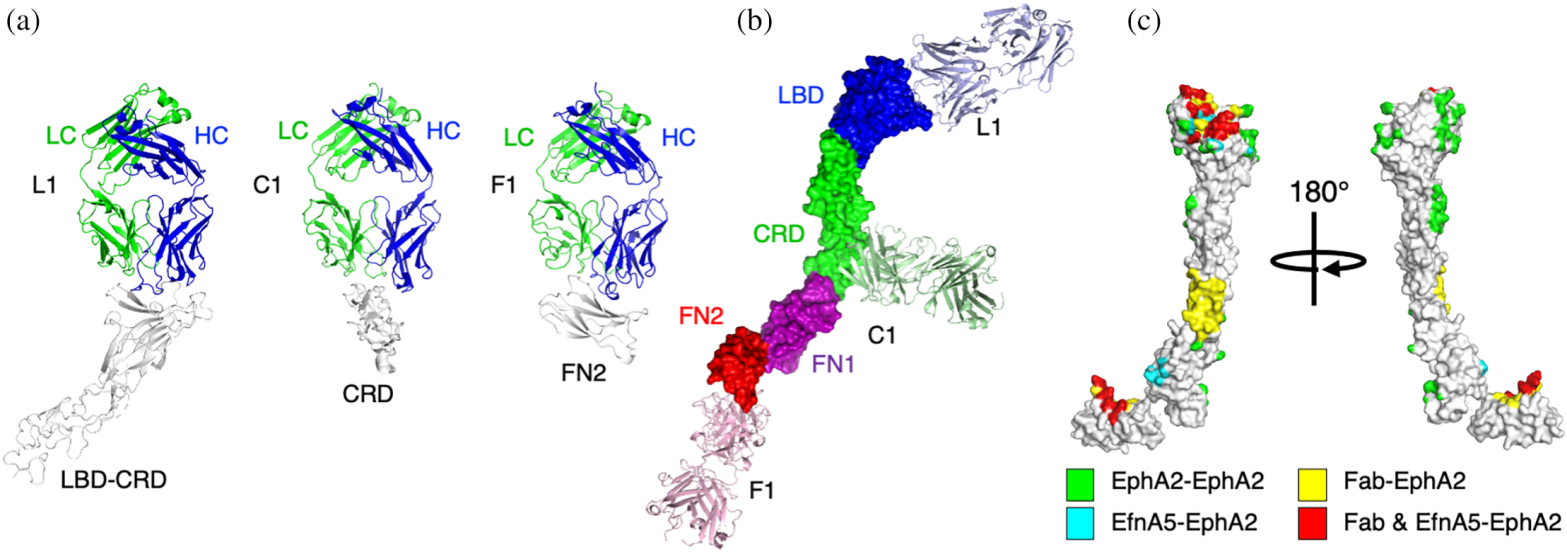
Structures of antigen‐binding fragments (Fabs) in complex with EphA2 domains. (a) Structures of Fabs L1, C1, and F1 in complex with EphA2 ligand‐binding domain (LBD) (*left*), cysteine‐rich domain (CRD) (*middle*), or C‐terminal fibronectin‐3 domain (FN2) (*right*), respectively. The main chains are shown as ribbons colored as follows: EphA2, gray; Fab heavy chain (HC), blue; Fab light chain (LC), green. (b) Superposition of the three Fab:EphA2‐domain structures with the structure of the full‐length EphA2 extracellular domain (ECD) (protein data bank (PDB) entry 3FL7). A single EphA2 ECD monomer is shown as a surface colored as follows: LBD, blue; CRD, green; N‐terminal fibronectin‐3 domain (FN1), purple; FN2, red. Fabs are shown as ribbons colored as follows: L1, light blue; C1, light green; F1, light red. (c) Intermolecular interfaces mapped onto the structure of the EphA2 ECD (PDB entry 2X11). The EphA2 ECD is shown as a gray surface, except regions involved in intermolecular interactions, which are shown in distinct colors. Regions that interact only with other EphA2 ECDs, EfnA5 ligand, or Fabs are colored green, cyan, or yellow, respectively. Regions that interact with both EfnA5 and Fab L1 (N‐terminal region) or F1 (C‐terminal region) are colored red.

Using optimized Fab frameworks, we solved the structures of Fabs L1, C1, and F1 in complex with the LBD‐CRD (Figure 3a, left), CRD (Figure 3a, middle), or FN2 (Figure 3a, right) ECD domains of EphA2 at 2.6, 1.9, or 4.2 Å resolution, respectively. Superposition of the structures onto the structure of the apo full‐length EphA2 ECD clearly showed how each Fab binds to a distinct region of the receptor, with Fab L1 binding to the N‐terminal LBD, Fab C1 binding to the central CRD, and Fab F1 binding to the C‐terminal FN2 region proximal to the single‐pass TM region (Figure 3b).

We also analyzed the superpositions to compare the EphA2 epitopes targeted by the Fabs to the intermolecular interfaces formed by EphA2 in signaling complexes (Figure 3c). The Fab L1 epitope does not overlap with either the LL or LL′ interfaces, leaving EphA2 self‐association unimpeded. Instead, the Fab L1 epitope overlaps significantly with Efn Site 1, consistent with the antagonist activity of the Fab due to steric competition. The Fab C1 epitope resides in the EGF subdomain of the CRD and does not overlap with the CC interface, which resides in the sushi subdomain of the CRD (Himanen et al., 2010; Seiradake et al., 2010), consistent with a lack of antagonist activity for Fab C1 (Figures 2e and 3c). The Fab F1 epitope overlaps significantly with Efn‐binding Site 2, suggesting that the antagonist activity of Fab F1 is, in part, due to interference with zippering of anti‐parallel EphA2‐Efn signaling assemblies (Figure 3c).

### 2.4 Structural basis for the high affinities of the anti‐EphA2 Fabs C1 and F1

While recognition of LBD by Abs has been characterized in previous studies (Himanen et al., 2009, 2010; Seiradake et al., 2010, 2013; Singh et al., 2018; Xu et al., 2013), engagement modes of Abs targeting the CRD and FN2 have yet to be elucidated. To determine the structural basis for high affinity, we examined the details of the molecular interactions of the C1 and F1 Fabs with EphA2.

**FIGURE 4:**
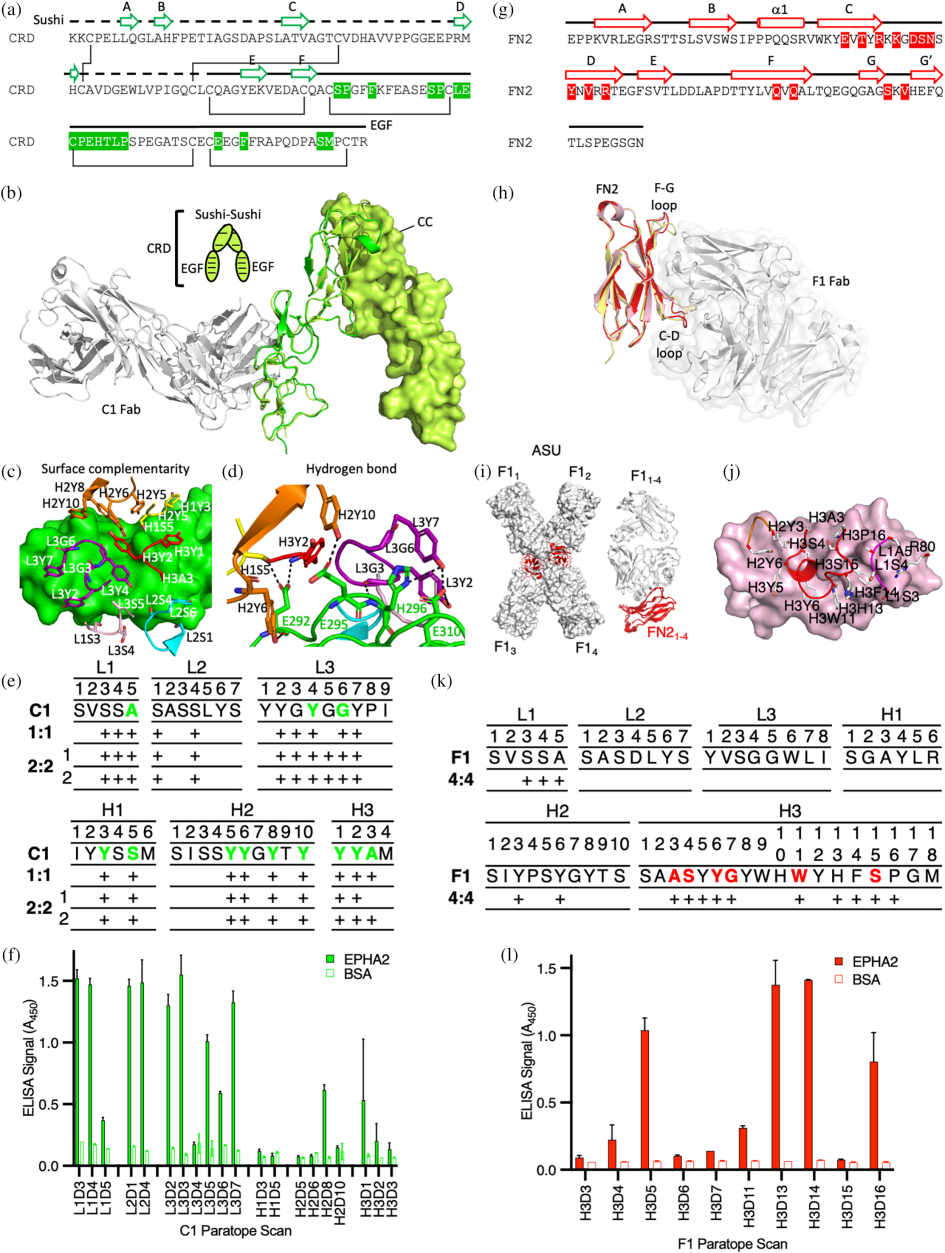
High affinity binding to the cysteine‐rich domain (CRD) and C‐terminal fibronectin‐3 domain (FN2) of EphA2. (a) Epitope contact residues (green) mapped on the sequence of the EphA2 CRD domain (K199‐P329). Disulfide bonds are illustrated with black lines. β‐Strand structure is illustrated as arrows. (b) Structure of antigen‐binding fragment (Fab) C1 (gray) bound to CRD (green) with the CC interface occupied by a second CRD protomer (yellow). Fab and CRD are illustrated as ribbons, and dimerized CRD is shown as a yellow surface. (c) Surface rendering of the CRD (green) with paratope residues of Fab C1 within 4.5 Å of the CRD illustrated as sticks and colored as follows: complementarity determining region (CDR)‐H1 (yellow), CDR‐H2 (orange), CDR‐H3 (red), CDR‐L1 (pink), CDR‐L2 (cyan), CDR‐L3 (purple), framework (white). (d) The hydrogen bond (dashed lines) network made between the paratope residues (CDRs colored as in c) and polar residues on the surface of the CRD (green) is illustrated for contact residues (sticks). (e) Antibody (Ab) paratope tables of CDRs for the C1 Ab. Residues with large contributions to the energetics of binding are bolded in green in the table. Residues observed in contact with EphA2 in distinct crystallized complexes are depicted (+). (f) Single point enzyme‐linked immunosorbent assay (ELISA) of Asp‐scanned paratope variants (100 nM) encompassing all observed contact residues to EphA2‐His (solid) or bovine serum albumin (BSA) (empty). Standard deviation *n* = 2. (g) Epitope contact residues (red) mapped on the sequence of the EphA2 FN2 (E438‐L527). β‐strand and α‐helical structures are illustrated as arrows or cylinders, respectively. (h) Structure of Fab F1 (gray ribbon) bound to FN2 (red) superimposed on the FN2 structure when bound to EfnA5 (pink, protein data bank (PDB) entry 2X11) or the apo state (yellow, PDB entry 3FL7). (i) Surface rendering of the four F1 Fabs (white) in complex with four FN2 domains (red ribbons) in the asymmetric unit (ASU) (*left*). Superimposed F1 Fab‐FN2 complexes (*right*). (j) Surface rendering of the FN2 (light pink) with paratope residues of Fab F1 within 4.5 Å of the CRD illustrated as sticks and colored as in (c). (k) Antibody paratope tables of CDRs for the F1 Ab. CDR‐H3 residues with large contributions to the energetics of binding are bolded in red in the table. Residues observed in contact with EphA2 in the crystallized complex are depicted (+). (l) Single point ELISA of Asp‐scanned CDR‐H3 variants (100 nM) binding to EphA2 (solid) or BSA (empty). Standard deviation *n* = 2.

The sushi and EGF subdomains that form the CRD are stabilized by two or four disulphides, respectively, but lack α‐helical and extensive β‐sheet secondary structure (Figure 4a). The epitope engaged by Fab C1 is located entirely within the EGF subdomain, which is partially conserved across human Eph receptors but diverse enough to create selectivity for the Fab (Figure S3A,B). Overlay of Fab C1 onto the dimer of EphA2 mediated by the CC interface revealed an engagement mode roughly perpendicular to the EphA2 CRD fold and distal from the CC interface formed by the sushi domains (Figure 4b). Fab C1 used a “lock‐and‐key” form of engagement where little to no conformational change in the EGF domain was necessary for binding. Analysis of the paratope revealed a minimalist synthetic surface composed of just four types of residues: Tyr, Ser, Ala, and Gly (Figure 4c). Notably, the Fab C1 paratope was predominantly formed by tyrosine residues, allowing for exquisite surface complementarity to the EGF domain with all six complementarity determining regions (CDRs) of the Fab contributing contacts. Engagement of the C1 paratope centers CDRs L3 and H3 on the EGF domain, allowing hydrogen bond contacts to form at the core of the paratope to four critical charged amino acids on the surface of the EGF fold (Figure 4d). This core set of polar contacts surrounded by van der Waals contacts of planar residues forms a highly favorable protein–protein interface. Unexpectedly, much of the buried surface area arose from undiversified CDRs (L1 and L2) in our Fab phage library. To assess the energetic contributions of the observed contact residues, we scanned the positional contacts with Asp, a residue not encoded in our library (Persson et al., 2013) and that minimized aggregation of the mutated paratopes (Dudgeon et al., 2009). Single point ELISA of the CDR variants mapped the residues most sensitive to Asp substitution, which we used as a proxy for positional contributions to energetics of binding (Figure 4e,f) (Pal et al., 2003). From this analysis we concluded that residues from diversified CDRs (L3, H1, H2, and H3) contributed the majority of the binding energetics. Only one contact from the undiversified CDRs (L1A5) contributed to the energetics of binding of EphA2 in our screen.

Examination of the Fab F1 interaction revealed an epitope on FN2 predominantly formed by the C‐D and F‐G strands and loops of the fibronectin type III fold (Figure 4g). The epitope is also partially conserved across human Eph receptors, but again, is diverse enough to create selectivity for the F1 paratope (Figure S3C–E). Superposition of the FN2 domains in the apo form (PDB entry 2X10), bound to EfnA5 (PDB entry 2X11), or bound to Fab F1 revealed subtle conformational adaptations of the C‐D and F‐G loops (Figure 4h). Though limited by the low resolution of the structure, a simple omit map at the core of the interface suggested that our model was well matched to the density (Figure S3F) and alignment of all four complexes revealed nearly identical engagement angles for each complex (r.m.s.d < 0.9 Å) (Figure 4i). In each complex, the observed interface was dominated by residues from CDR‐H3 with minor contacts from CDR‐H2 and CDR‐L1 (Figure 4j). To further validate our findings, we performed an Asp scan (as described above) of the dominant CDR‐H3 of the F1 paratope, which revealed a six‐residue hotspot signature that contributed to the energetics of binding, consistent with the structural analysis (Figure 4k,l). Sequence analysis of the natural paralog sequence variation revealed that F1 engaged a highly diverse pocket of Eph isoforms using the energetic hotspot residues (Figure S3D). Together, these data help to explain the exclusive specificity of F1 for EphA2.

### 2.5 Structural basis for the high affinity and specificity of anti‐EphA2 Fab L1

**FIGURE 5:**
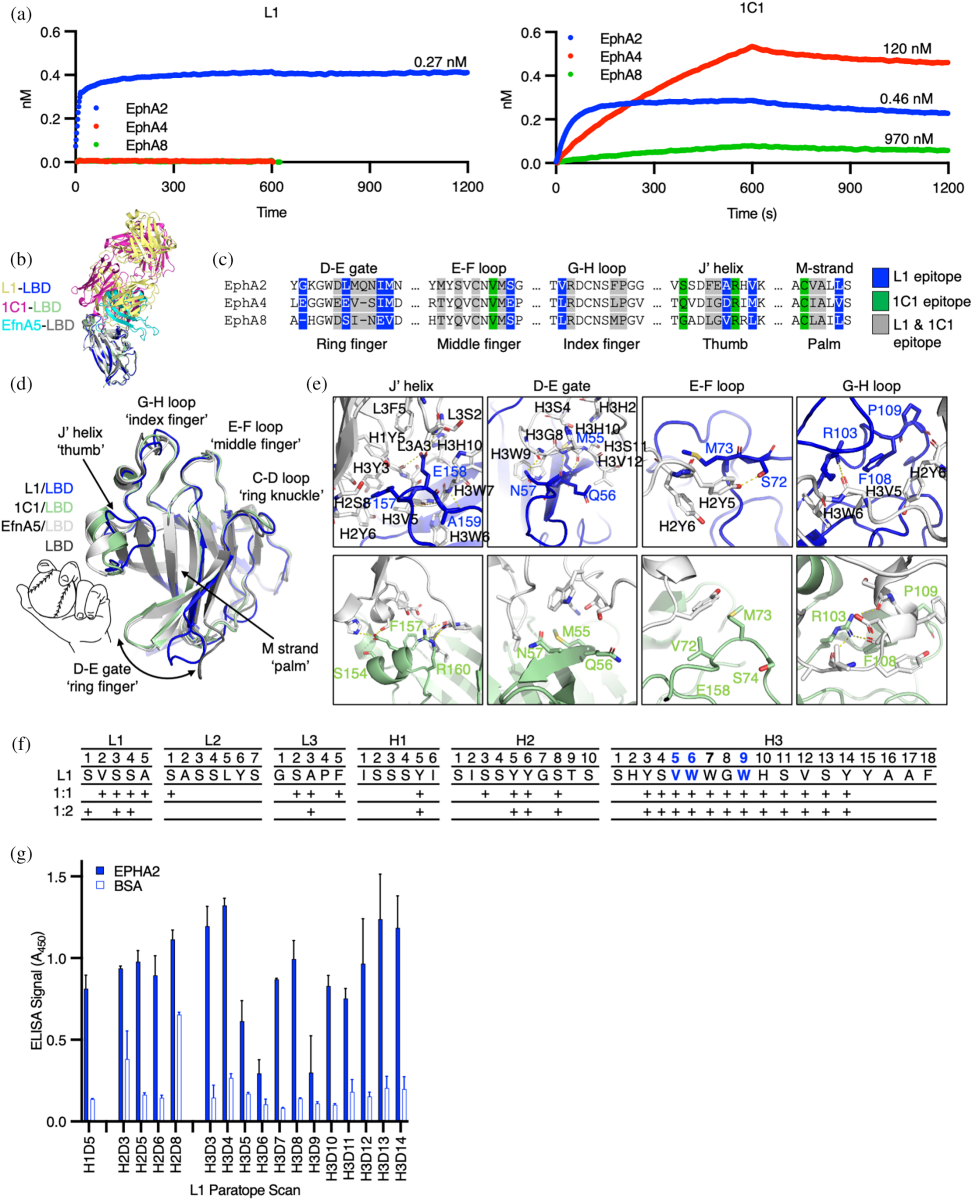
Molecular basis for the absolute specificity of antibody (Ab) L1 for the EphA2 ligand‐binding domain (LBD). (a) Biolayer interferometry sensorgrams of 300 nM EphA2‐Fc (blue), EphA4‐Fc (red), or EphA8‐Fc (green) binding to immobilized IgG L1 (left) or 1C1 (right). (b) Structural alignments of the L1:LBD complex (yellow/blue) with the EfnA5:LBD (cyan/gray) and 1C1:LBD (pink/green) complexes superimposed. (c) Epitope contact residues to L1 (blue), 1C1 (green), or both (gray) mapped on the sequence of the EphA2 LBD. (d) Overlays of the following EphA2 structures: antigen‐binding fragment (Fab) L1:LBD‐cysteine‐rich domain (CRD) complex (blue), Fab 1C1:LBD complex (green, protein data bank (PDB) entry 3SKJ), apo LBD (dark gray, PDB entry 3C8X), and EfnA5:LBD complex (light gray, PDB entry 2X11). (e) Comparison of contact residues at the interfaces between the LBD (blue) and Fab L1 (gray, *top*) or LBD (green) and Fab 1C1 (gray, *bottom*). Contact residues (<4.5 Å) are shown as sticks on ribbons. Hydrogen bonds are shown as dashed lines. (f) Paratope table of complementarity determining regions (CDRs) for L1 Abs. Residues with large contributions to the energetics of binding are bolded in blue in the table. Residues observed in contact with EphA2 in distinct crystallized complexes are depicted (+). (g) Single point enzyme‐linked immunosorbent assay (ELISA) of Asp‐scanned paratope variants (100 nM) binding EphA2 (solid) or bovine serum albumin (BSA) (empty). Standard deviation *n* = 2.

The LBD domain sequences across the Eph family are highly conserved, making it challenging to engineer Abs with absolute specificity for individual family members (Figure S4A). To evaluate specificity, we compared the binding of IgG L1 to that of the agonist IgG 1C1, a benchmark clinical EphA2 Ab (Peng et al., 2011), to the full complement of human Eph ECDs (Figure S4B,C). We observed exclusive and tight binding of IgG L1 to the EphA2 ECD (*K*_D_ = 0.27 nM), while IgG 1C1 bound tightly to EphA2 (*K*_D_ = 0.46 nM) but also showed weak affinity for EphA4 (*K*_D_ = 120 nM) and EphA8 (*K*_D_ = 970 nM) (Figure 5a). Both L1 and 1C1 targeted the same binding pocket of EphA2 recognized by EfnA5 and largely overlap in their molecular interactions, but only L1 proved to be absolutely selective for EphA2 (Figure 5a–c).

We then examined the interactions between paratopes and epitopes to determine the molecular basis for the exquisite specificity of Fab L1 for EphA2 and the more promiscuous specificity of Fab 1C1. Mapping of epitopes on EphA2 revealed that L1 and 1C1 bind similar regions of the LBD (Figure 5c). The recognition surface of EphA2 LBD resembles a left‐handed “fastball grip” where contacts are made by the J′ helical region (thumb), G‐H loop (index finger), E‐F loop (middle finger), C‐D loop (ring knuckle), and D‐E gate (ring finger) (Figure 5d). Overlays of the L1‐LBD complex and 1C1‐LBD complex onto apo and Efn‐bound forms of EphA2 revealed that two regions exhibit considerable conformational diversity. The region of highest conformational diversity was the D‐E ring finger, which swings from open (apo) to closed (Efn‐bound) forms across the structures, displacing the loop by as much as 15 Å to “grip” ligands in the binding pocket. In the L1‐LBD complex, the D‐E ring finger is swung open, allowing close interactions between Fab L1 and the M‐strand “palm” of the binding pocket. In contrast, in the 1C1‐LBD complex, the D‐E gate is swung by 10 Å to a more closed position. The other notable site of conformational diversity is the J′ helical thumb. Engagement of Fab 1C1 induces a small shift of the J′ helix to engage the heavy chain variable region (V_H_) of 1C1 (Figure 5d,e). In contrast, L1 engagement completely unravels the helix to form an extended loop that juts into a binding cavity created by the light chain variable region (V_L_) to specifically coordinate LBD residues E^158^ and A^159^ with hydrogen bonds while burying F^157^ in a hydrophobic pocket formed by the V_L_ (Figure 5d,e). Critically, the F^157^‐E^158^‐A^159^ contact triad of the thumb is exclusive to EphA2 and is distinct from the corresponding triads in EphA4, EphA8, and all other Eph isoforms (Figures 5c and S4A). Though overlapping in epitope, comparison of L1 and 1C1 contacts thus revealed that each Fab binds a distinct conformation of the LBD (Figure 5b–d). Notably, Fab L1 makes more main chain hydrogen bonds, indicative of high surface complementarity between the epitope and paratope (Figure 5e). The molecular determinants enabled by a distinct conformation of the LBD therefore drive the high affinity and specificity of Fab L1 for EphA2.

Structural characterization of Fab L1 resulted in two crystal forms (Table S1). Across these two structures, we observed many shared heavy chain contacts responsible for LBD recognition, while the light chain contacts were less consistent across the two models (Figure 5f). To determine which residues contribute significantly to the binding energetics, we scanned the heavy chain contacts of the Fab L1 paratope and found that all but 3 amino acids could be substituted with minimal changes in ELISA signal (Figure 5f,g). We therefore found a very small energetic “hot spot” for Fab L1 recognition, despite having a long CDR‐H3 buried in the LBD pocket.

### 2.6 IgG L1: A complete agonist of EphA2 signaling

**FIGURE 6:**
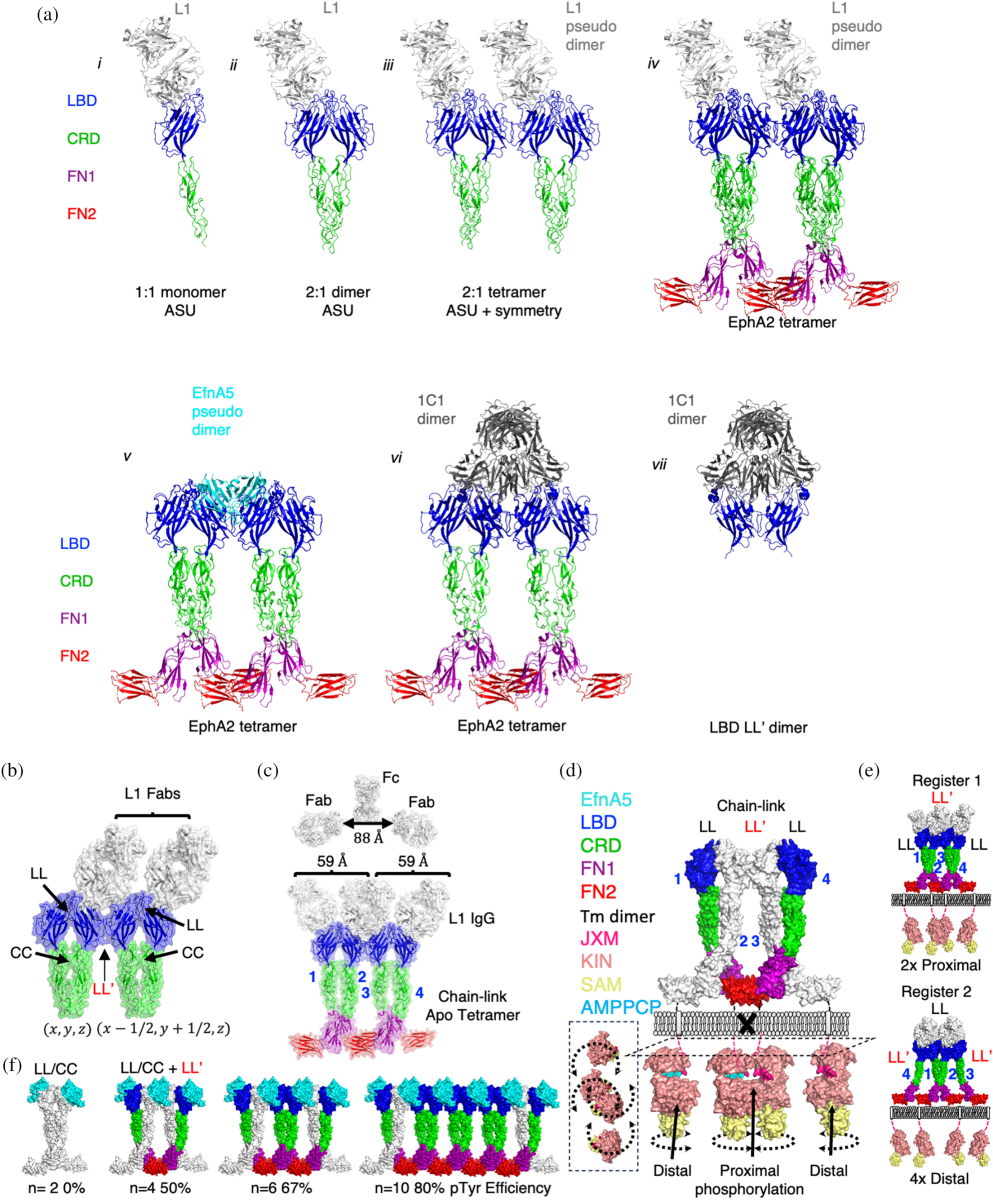
Activation of EphA2 through chain‐linked clusters. (a) Common receptor arrangements observed in crystal structures of EphA2 bound to agonist ligands. The structures of the 1:1L1:ligand‐binding domain (LBD)‐cysteine‐rich domain (CRD) (*i*), 1:2L1:LBD‐CRD (*ii*), 1:2L1:LBD‐CRD symmetrical dimer (*iii*), the L1 dimer superimposed onto the chain‐link cluster (protein data bank (PDB) entry 3FL7) (*iv*), the chain‐link cluster superimposed with EfnA5:EphA2 (PDB entry 3FL7/3MX0) (*v*), the chain‐linked cluster superimposed with the 1C1 symmetry dimer (PDB entry 3FL7/3SKJ) (*vi*) and the 1C1:LBD dimer (PDB entry 3SKJ) (*vii*) identified by analyzing symmetrically related molecules in the unit cell are shown as ribbons. Antigen‐binding fragments (Fabs) are illustrated as gray, ephrin (Efn) as cyan and the domains of EphA2 are illustrated as follows: ligand‐binding domain (LBD) (blue), cysteine‐rich domain (CRD) (green), N‐terminal fibronectin‐3 domain (FN1) (purple) and C‐terminal fibronectin‐3 domain (FN2) (red). (b) Surface rendering of the chain‐link cluster observed in the 1:2 complex of Fab L1:LBD‐CRD where the EphA2 fragments are dimerized in the asymmetric unit (ASU) by LL (blue) and CC (green) interfaces and interact through LL′ interfaces between symmetrically related molecules. (c) Surface rendering of human IgG (adapted from PDB entry 1HZH) with distance between Fabs annotated (*top*). Overlay of four L1 Fabs (representing two IgGs) onto the tetrameric chain‐link cluster of EphA2 observed in the apo complex (PDB entry 3FL7) (*bottom*). Surfaces are colored as follows: Fabs and IgGs (white), LBD (blue), CRD (green), FN1 (purple), and FN2 (red). (d) Profile surface rendering of EphA2 receptor clusters induced by IgG L1, using structures of the EphA2 extracellular domain (ECD) and intracellular domain (ICD) (PDB entries 3FL7 and 7KJA). ECDs of EphA2 that recruit proximal kinases are colored, whereas ECDs of clustered EphA2 that give rise to distal kinases are depicted in white. Domains of EphA2 are colored as indicated on the left. Domains responsible for LL (black) and LL′ (red) clustering are indicated in dashed boxes. Inset is a top‐down perspective of the ICDs alone. (e) Full receptor models of two potential tetramer registers recruited by L1 IgGs. Register 1 (*top*) and register 2 (*bottom*) EphA2 tetramers form distinct recruitment profiles for intracellular kinases, resulting in proximal or distal recruitment, respectively. Two L1 IgGs are illustrated as four Fab arms (white). (f) Profile surface renderings of chain‐link clusters of increasing size (left to right) with predicted efficiency of pTyr^588^ for each cluster shown below. Protomers resulting in distally recruited kinases are colored white. Efficiency percentages represent the fraction of kinases predicted to be phosphorylated per cluster size illustrated.

Given that IgG L1, IgG 1C1, and EfnA5‐Fc share the same epitope, we anticipated that each molecule agonized EphA2 by inducing similar signal‐competent clusters. We therefore analyzed the lattices of each agonist complex to determine if there were structural arrangements shared by each. The crystal structure of Fab L1 bound to LBD‐CRD revealed the location of the epitope on the EphA2 LBD (Figure 6a, *i*), but lacked Eph‐Eph interfaces associated with clustering and signaling. Utilizing an alternative Fab framework optimized for crystallization (Bruce et al., 2023, 2024), we were able to capture an additional crystal lattice packing arrangement that revealed the anticipated higher order protomer complex of Fab L1 and LBD‐CRD (Figure 6a, *ii*), albeit at a lower resolution of 4.6 Å. Molecular replacement of the lattice revealed a structure with one Fab L1 bound to two LBD‐CRD molecules with a solvent content of 65%. In this structure, a single Fab engaged an LL/CC dimer of EphA2 in the asymmetric unit (ASU) as observed in all closed forward‐signaling‐competent structures (Figures 1a,c and S1B). Comparison of symmetrically related molecules in the L1 and 1C1 Fab complexes (PDB entry 3SKJ) identified the same EphA2 LBD chain‐link self‐association pattern as observed in the closed tetrameric EphA2 complexes (PDB entries 3FL7, 3MXO, 3MBW) (Figure 6a, *iii–vii*). These data demonstrate that both agonist IgGs and recombinant Efn‐Fc molecules engaging the Efn‐binding pocket of LBD can trigger EphA2 signaling by stabilizing similar tetrameric EphA2 arrangements (Figures 1e and S1B).

We then asked if EphA2 was able to form clusters through its LL and LL′ interfaces in the presence of Fab L1. Evaluation of the symmetrically related molecules revealed an LL′ interface critical to forming this lattice of symmetrically related complexes, resulting in a 2:4 Fab‐EphA2 biological unit (Figure 6b). Using the structure of a full human IgG1 (PDB entry 1HZH) as a benchmark for IgG reach (88 Å), we determined that the observed span of 59 Å between L1 Fabs was compatible with IgG co‐engagement (Figure 6c). We then further modeled the anticipated 4:4 complex at Ab saturation by overlaying our 1:1 protomer model (Figure 6a,i) onto the apo tetramer (PDB entry 3FL7) (Figure 6c). This model demonstrated that two L1 IgGs could potentially bind the four receptors in the closed tetramer without steric clashes. Therefore, we concluded that L1 IgGs induce the same cross‐linked tetravalent cluster as is achieved by Efns, by stably bridging a dimer (LL′) of dimers (LL/CC) with either one or potentially two L1 IgG co‐engagements.

To more fully understand the functional consequences of EphA2 clustering stoichiometry, we created a theoretical model of the full‐length EphA2 by approximating the positions of the four protomers of the SAM domains of the ICDs (PDB entry 7KJA) relative to the FN2 domains. While signal‐competent dimers of RTKs recruit their kinases with a TM proximity of <40 Å (Figure S5A), static models of EphA2 tetramers recruit their kinases with a TM proximity of 57–60 Å (Figure 1a); a marked deviation from other signal‐competent RTK dimers, despite the kinase domains being equivalent in size (Figure S5B,C). It has also been shown that TM dimerization is critical for the production of pTyr^772^ modifications of EphA2 (Alves et al., 2018) as observed for two of our agonist IgGs and Efn‐Fc (Figure 2i and S2H,I). In silico evaluation of the closed tetramer revealed that the static FN2 positions in the crystal structures were too distal from each other to allow TM dimerization or kinase recruitment. We therefore conclude that static models of EphA2 tetramers do not completely represent the active state of the ECD conducive to phosphorylation of the ICD. We then considered a dynamic model of EphA2 where TM and kinase positioning was not fixed but could pivot on the FN1‐FN2 hinge to create the proximity for TM dimerization (Figure 6d) (Alves et al., 2018). With this consideration, kinases linked to EphA2 ECD protomers 1 and 4 could approach each other resulting in productive engagement of TM dimers and recruited kinases, similar to other RTKs. Protomers 2 and 3, however, remained distal even if dynamic, suggesting that tetravalent EphA2 clusters could preferentially trans‐phosphorylate the internal reciprocal and proximal recruited kinases at their juxtamembrane, whereas distal kinases would remain unphosphorylated. Unlike Efns that contribute cross‐linking contacts to tetrameric assembly, tetramers bridged by IgGs could potentially give rise to two registers of EphA2 tetramers. In register 1, LL′ cross‐linking of LL/CC dimers promotes proximal recruitment of kinases, whereas in register 2, the LL/CC cross‐linking of LL′ dimers places all four kinases distal from each other (Figure 6e). We therefore associated register 1 tetramer formation to the minimal signaling‐competent cluster, as register 2 tetramers required another condensation of preformed dimers (i.e., a hexamer) to create the first proximal and reciprocal kinase recruitment.

Importantly, the tandem LL and LL′ interfaces observed in forward signaling complexes enable EphA2 protomers to further polymerize as was observed in the 1:2 L1‐EphA2 lattice (Figure 6b). In silico models of polymerized Efn‐EphA2 tetramer clusters were built by modularly adding EfnA5‐EphA2 dimers in a chain‐link array (Figure 6f). We noted that the proportion of proximal and reciprocal kinase recruitment improved as EphA2 clusters polymerized to greater than four protomers, suggesting that larger clusters of EphA2 can more efficiently signal through pTyr^588^ relative to smaller clusters of equal protomers, consistent with the observed correlations between Efn‐mediated cluster size and signal strength (Schaupp et al., 2014; Zapata‐Mercado et al., 2022). Thus, our modified dynamic tetramer model better represented the observed principles of cellular EphA2 triggering that could not be accounted for by dimer triggering models.

### 2.7 Fab F1: A complete antagonist of EfnA1‐mediated signaling clusters

**FIGURE 7:**
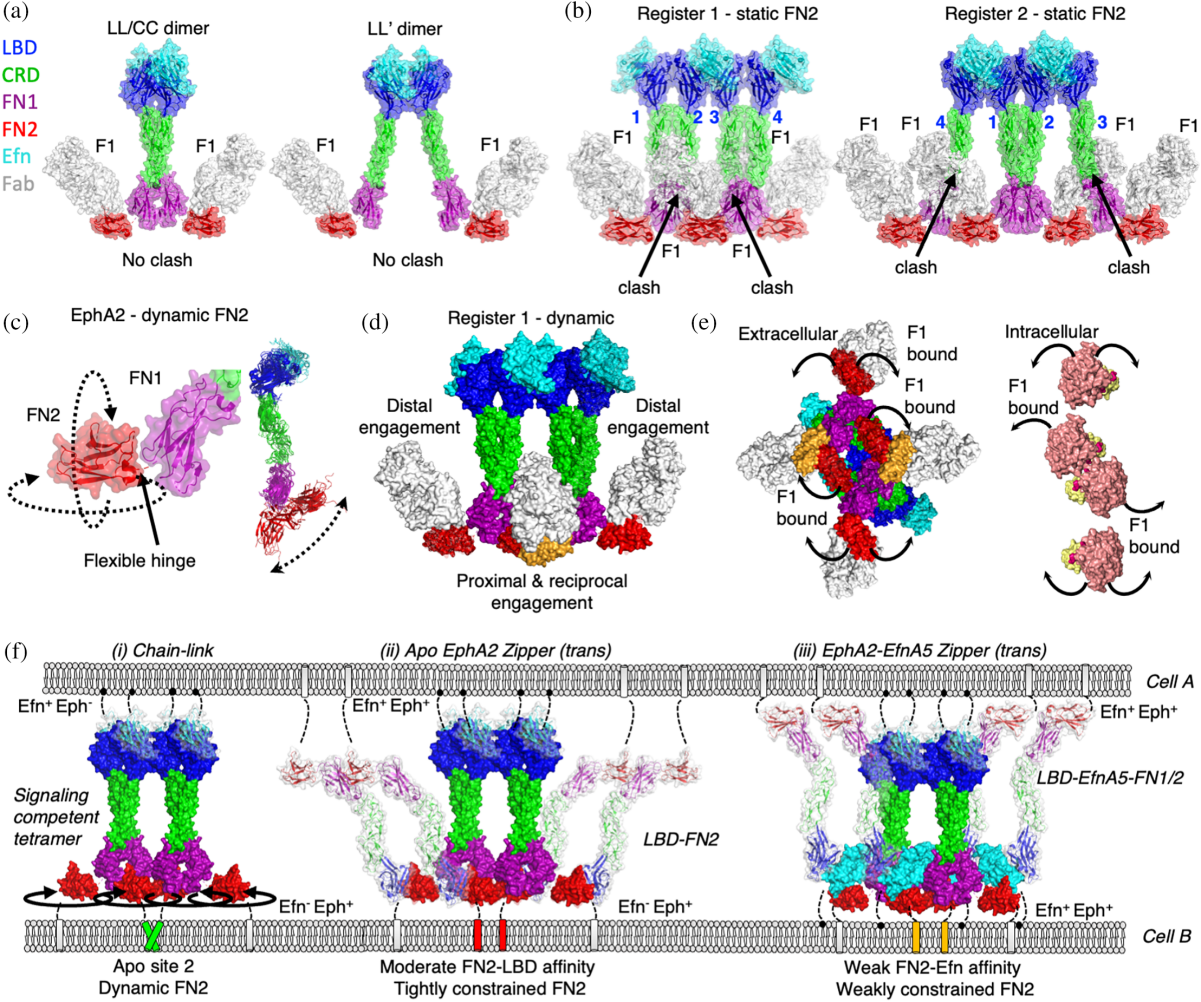
Uncoupling chain‐linked activation through constrained C‐terminal fibronectin‐3 domain (FN2) dynamics. (a) Superposition of F1:FN2 onto the LL/CC dimer (*left*) or the LL′ dimer (*right*) to evaluate steric clashes. (b) Superposition of F1:FN2 onto the chain‐link tetramer in register 1 (*left*) or register 2 (*right*) to evaluate steric clashes. Clashes are indicated with arrows. (c) Surface rendering of N‐terminal fibronectin‐3 domain (FN1)‐FN2 modular pair (adapted from protein data bank (PDB) entry 2X11) (*left*). FN1 (purple) and FN2 (red) are linked by a flexible polypeptide linker (indicated by arrow) whose density is missing in the crystal lattice. Flexibility of the hinge allows FN2 to adopt many conformations relative to FN1 as globally observed for structures of EphA2 (PDB entries 2X11, 3FL7, and 2X10) and its homolog EphA4 (PDB entries 4BK5, 4BKF, 4BK4, 4M4R, and 4M4P) when overlaid (*right*). (d) A theoretical dynamic model of rotated proximal and reciprocal FN2 domains (orange) constrained by F1 antigen‐binding fragments (Fabs) overlaid onto the observed active chain‐link cluster where FN2 domains (red) are illustrated (PDB entry 2X11). F1 Fabs (white) bound to EphA2 protomers recruiting distal kinases fail to sterically constrain the dynamics of FN2 and maintain the same dynamic properties as in activated EphA2 (red, PDB entry 2X11). Fab F1 engagement of EphA2 protomers engaging proximal and reciprocal protomers causes the FN2 to pivot (orange) on the flexible hinge away from the plane of the EphA2 cluster to avoid steric clashes between Fab F1 and the FN1 domains of adjacent receptors. (e) An open book projection from the membrane of F1‐displaced reciprocal and proximal FN2 domains of the extracellular domain (ECD) (*left*) and the F1‐induced kinase displacement of the intracellular domain (ICD) (*right*) of EphA2 chain‐link tetramers. Approximation of the conformational pivot of the ECD is illustrated as dashed arrows connecting apo FN2 (red) and F1‐bound FN2 (orange). The modeled pivot is projected onto the ICD kinases, illustrating the kinase displacement. ECD domains are colored as in (a). (f) Models of possible natural signal attentuation through FN2 constraint during cellular EphA2 zippered synapses. Surface representations of EphA2 in (*i*) a static signaling‐competent cluster (PDB entry 2X11), (*ii*) an auto‐inhibited cluster (PDB entry 2X11 and 3FL7), and (*iii*) an Eph‐Efn zipper (PDB entry 2X11) are illustrated in trans configuration. EphA2 and EfnA5 receptors on the surface of cell A are illustrated as colored ribbons with a white surface. EphA2 and EfnA5 receptors on the surface of cell B are illustrated as colored surfaces according to domains as in (a). Dynamic FN2 domains are illustrated with dashed circled arrows.

To better understand the mechanisms of Efn‐mediated signaling clusters, we evaluated the modes of action of the two complete antagonists, Fabs L1 and F1. Like most EphA2 antagonists, Fab L1 bound to the LBD and significantly overlapped with the high‐affinity Efn‐binding Site 1, demonstrating a simple Efn‐competitive mechanism of inhibition (Figures 3c and 5b). However, the mode of action for Fab F1 was less obvious. Thus, we asked if the inhibitory activity of Fab F1 was driven by occlusion of EphA2‐EphA2 dimer interfaces required for kinase recruitment. To evaluate this, we superposed Fab F1 onto LL/CC or LL′ dimeric structures of EfnA5‐EphA2 (Figure 7a). We observed no steric clashes with either dimer, suggesting that neither dimer is occluded when bound to F1. This analysis appeared to support our proposition that dimers of EphA2 were not in themselves the structural arrangement responsible for EphA2 Tyr phosphorylation. We then asked if Fab F1 could sterically block tetrameric clusters of EphA2 by overlaying the structure onto models of EphA2 tetramers in register 1 or 2 (Figure 7b). Both registers of the EphA2 tetramer gave rise to steric clashes between FN1 and Fab F1 engaged to protomers responsible for proximal and reciprocal kinase recruitment (protomers 1 and 4 in register 1), while engagement to distal kinases created no steric clashes. These overlays revealed a potential mechanism of non‐competitive inhibition and further supported our hypothesis that tetramerization of EphA2 is necessary for triggering of EphA2 signaling.

The FN2 domain represents a particularly malleable region of Eph receptors owing to the flexible hinge region connecting FN1 to FN2, which adopts many diverse conformations across structures of EphA2 and EphA4 (Figure 7c). We therefore considered Fab F1 binding in the context of chain‐link clusters with dynamic FN2 positioning in silico (Figure 7d,e). Engagement of Fab F1 to distal protomers 2 and 3 of register 1 tetramers would have no impact on distal kinase positioning, whereas Fab F1 engagement of proximal protomers 1 and 4 could constrain the conformations allowed by the FN1‐FN2 hinge, forcing this region of the receptor to pivot away from the plane of the receptor cluster (Figure 7d,e). This in turn would displace only the core, proximal, and reciprocal kinases from each other and would thus interfere with trans‐phosphorylation of pTyr^588^ (Figure 7e). Our mechanistic model for F1 Fab inhibition therefore inferred that dynamic FN2 positioning was an essential feature of the EphA2 active state induced by Efn‐mediated tetramers (Figure 7f).

We then considered other protein–protein interactions mediated by FN2 and their potential roles in modulating FN2 dynamics and signaling. Contact surfaces within crystal structures of apo EphA2 and EphA4 have been used as evidence for head‐to‐tail protomer assemblies that create auto‐inhibitory auxiliary zones in EphA2 clusters with enhancing non‐canonical signaling (Shi et al., 2023). In this structural model, apo EphA2 engages a region of FN2 that overlaps with the F1 epitope through its LBD to form a head‐to‐tail arrangement (Figure S6A). Similar to the F1 Fab, head‐to‐tail engagement in cis or trans could limit FN2 dynamics to form an auto‐inhibited state (Figure 7f). However, occlusion of the head‐to‐tail engagement by the F1 Fab, F1 IgG, or F1 DFF did not appear to influence non‐canonical signaling in BxPC3 or MDA MB231 (Figures 2i and S2H,I). Comparatively, contact surfaces within crystal structures have been used as evidence for trans‐zippering of EfnA5/EphA2 complexes to form a distinct active state (Seiradake et al., 2010). This is achieved through weak EfnA5 engagement to Site 2 of FN2, a region that significantly overlaps with the F1 epitope and is responsible for auto‐inhibition of the receptor (Figure S6A,B). *In silico* models of these zippered complexes revealed that dynamics of FN2 would also be dependent on the weak affinity for EfnA5 to Site 2, potentially resulting in an intermediate active state compared to fully active chain‐linked clusters and auto‐inhibited chain‐linked clusters (Figure 7f). We therefore concluded that Fab F1 attenuates EphA2 signaling by engaging a natural auto‐inhibitory region at Site 2, but avid engagement of this same site was capable of weak‐partial or partial agonism depending on the avidity of the Ab modality.

### 2.8 IgG C1: A partial agonist of EphA2 signaling

**FIGURE 8:**
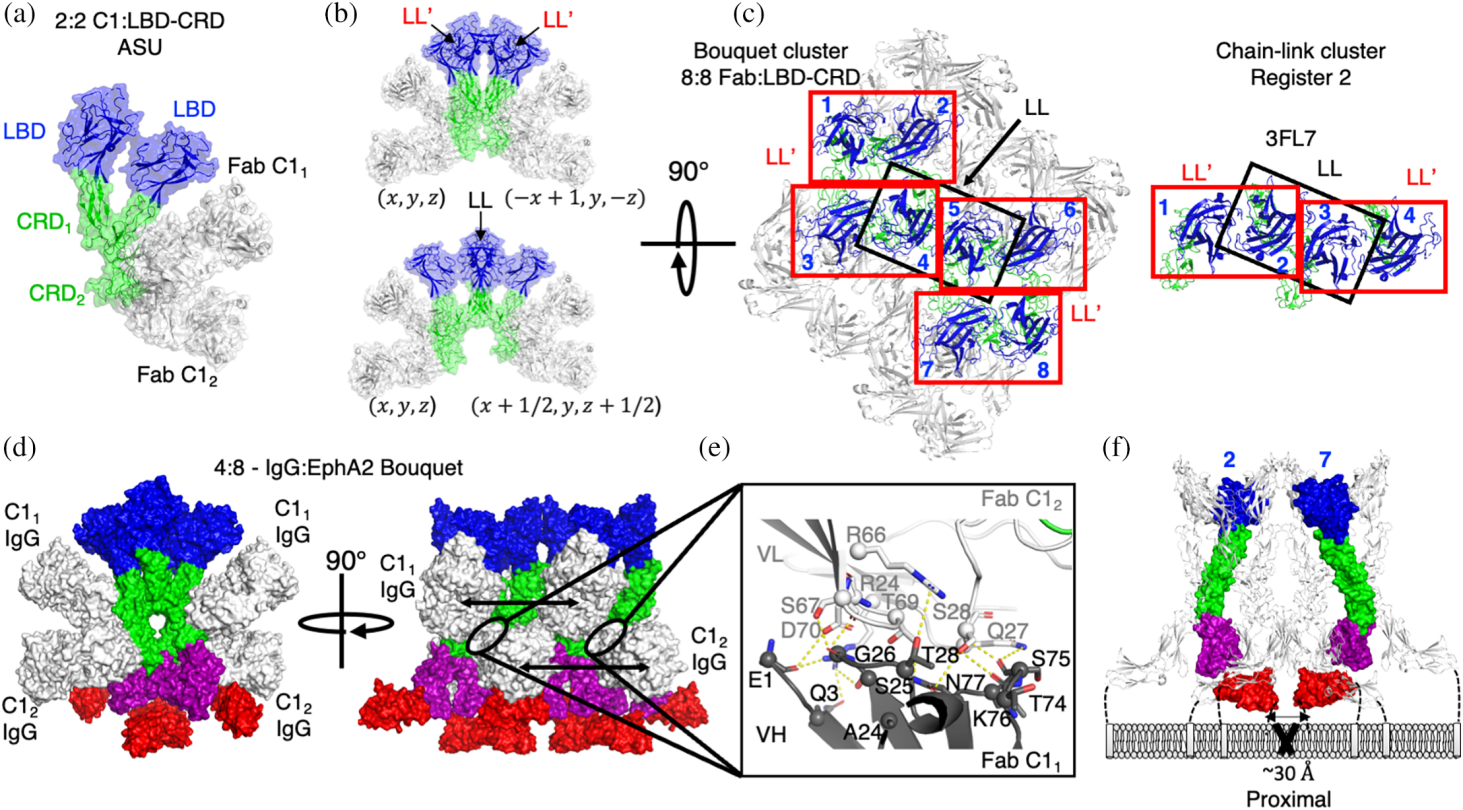
Partial agonist bouquet clusters. (a) Surface rendering of the asymmetric unit (ASU) of the 2:2 antigen‐binding fragment (Fab) C1:ligand‐binding domain (LBD)‐cysteine‐rich domain (CRD) complex. Fabs (white) are denoted C1_1_ and C1_2_, whereas the protomers of EphA2 are denoted LBD‐CRD_1_ and LBD‐CRD_2_. Cysteine‐rich domains (CRDs) are colored green and ligand‐binding domains (LBDs) blue. (b) Surface rendering of symmetrically related complexes in ASU1 and ASU2 associated by LL′ interfaces between LBD‐CRD_1_ and LBD‐CRD_2_ (*top*). CRDs (green) from ASU1 and ASU2 are bound by Fabs (white) to form a bouquet of EphA2 “flowers” being held by four “hands.” Surface rendering of symmetrically related complexes in ASU1 and ASU3 associated by one LL interface between LBD‐CRD_1_ and LBD‐CRD_1_ (*bottom*). (c) Top–down perspective of the octamer Fab:EphA2 extracellular domain (ECD) complex condensed by eight Fab/4 IgGs containing both LL and LL′ interacting protomers (*left*) and the register 2 tetramer chain‐link cluster (*right*) (protein data bank (PDB) entry 3FL7). EphA2 ECDs and Fabs are colored as in (a). IgG linkages between C1_1_ and C1_2_ Fabs are indicated with arrows. (d) Model of the full C1:ECD complex of the octamer created by overlaying the full ECD of EphA2 (PDB entry 2X11) onto the 8:8 Fab‐C1:LBD‐CRD. EphA2 domains are colored as follows: LBD (blue), CRD (green), N‐terminal fibronectin‐3 domain (purple), C‐terminal fibronectin‐3 domain (red). (e) V_H_/V_L_ interface of EphA2‐bound Fabs/IgGs in the bouquet cluster. V_H_ (dark gray) and V_L_ (white) contact residues (<4.5 Å) are illustrated as sticks with Cα spheres. Hydrogen bonds are illustrated as dashed yellow lines. (f) The resultant proximal EphA2 pair with the bouquet octamer cluster is shown in domain colors. Distal receptors are shown as white ribbons.

Similar to Fab L1, our first structure of Fab C1 in complex with CRD (Figure 3a–c) failed to reveal any receptor interfaces that could explain the induced partial activation of EphA2 by IgG C1. Given the importance of the LBD in clustering EphA2, we solved the structure of Fab C1 bound to the LBD‐CRD fragment to 3 Å resolution (Figure 8a), which revealed an ASU containing two C1 Fabs and two LBD‐CRD. In this structure, the LBD‐CRD was observed in two conformations (Figure S7A), resulting in two distinct but overlapping paratopes and epitopes for the two Fabs (Figure S7B,C). The first Fab (C1_1_) interacts with both protomers of EphA2 (CRD_1_ and CRD_2_) through distinct contact surfaces. The second Fab (C1_2_) interacts exclusively with the second EphA2 protomer (CRD_2_) at the same epitope observed in the 1:1 complex (Figure 3a–c). Fab C1_1_ distinctly uses its V_L_ framework region to associate with the sushi domain of CRD_2_, thereby bridging the two EphA2 ECDs and their bound Fabs into a quaternary complex (Figure S7B,C). Importantly, this secondary engagement to the sushi domain occludes the CC interface surface of CRD_2_ necessary for conventional chain‐link and zipper cluster arrays.

We then asked if these C1_1_‐ and C1_2_‐bound EphA2 protomers still formed higher ordered clusters through the remaining accessible LL/CC and LL′ interfaces. Analysis of the symmetrically related molecules revealed a 4:4 Fab‐EphA2 tetramer where both LBD‐CRD_1_ and LBD‐CRD_2_ form homodimers with symmetrically related LBD‐CRD protomers through their LL′ interfaces to form a quaternary structure resembling a “bouquet” of four EphA2 ECDs held by four Fab C1 “hands” (Figure 8b, top). Likewise, we also observed an LL/CC mediated interaction connecting symmetrically related molecules of LBD‐CRD_1_ protomers that remained accessible in the bouquet cluster (Figure 8b, bottom). The bouquet clusters of EphA2 were therefore driven by the same interfaces observed in the chain‐link clusters (Figures 1a–c and 6a,b) but adapted due to the occlusion of the CC in CRD_2_.

We then compared the cluster arrangement of the 8:8 bouquet cluster to that of the tetramer chain‐link cluster and noted that the C1 bouquet cluster shared the same register 2 tetramer (signaling incompetent) at its core (Figure 8c). But due to bouquet formation, this cluster further recruited an additional four EphA2 protomers (Figure 8c). We modeled the resultant signaling cluster of EphA2 ECDs by superposing full‐length EphA2 ECD (PDB entry 2X11) onto the quaternary 8:8 C1 Fab‐LBD‐CRD arrangement that accounted for both the LL and LL'mediated interfaces in the crystal lattice (Figure 8d). These data revealed a partial agonist cluster of eight EphA2 protomers cross‐linked by as many as four C1 IgGs (Figure 8e). Here, Fabs C1_1_ and C1_2_ arrayed approximately equidistant from each other at 73 or 74 Å apart, respectively, each consistent with the reach of IgG1, creating some ambiguity as to the most probable IgG linkage of the observed Fabs. Given the more extensive buried surface of C1_1_ Fabs, we reasoned that avid engagement of these sites likely initiates partial agonist cluster formation, which is then further stabilized by the weaker engagement of avid IgG C1_2_ to form a 4:8 IgG–EphA2 protomer arrangement at saturation. Each set of LL′ bouquet tetramers is then bridged by IgG C1 in a +/− 4 protomer IgG register assembling octamer‐based EphA2 clusters. The octomer arrangement could be induced by as few as one and as many as four C1 IgG engagements. Interestingly, we observed a significant hydrogen‐bond‐rich interface between adjacent C1_1_ and C1_2_ through the frameworks of V_H_ and V_L_, respectively, suggesting that C1_1_ and C1_2_ IgGs also self‐associate upon binding EphA2 in a 4:8 IgG–EphA2 cluster (Figure 8e).

We then interrogated the anticipated proximity of the intracellular kinases by evaluating the distances and geometric placement of the FN2 domains within the modeled clusters. Similar to chain‐link clusters, we identified a single pair of receptors recruiting proximal kinases through protomer 2 and protomer 7 in the octamer complex (Figure 8f). The proximal kinase EphA2 protomers did not make direct contact with each other through their ECDs. Instead, recruitment of eight protomers of EphA2 was required to create a ternary complex that recruited these two protomers in proximity. As the number of protomers necessary for a proximal‐recruitment doubles compared to Efn or IgG L1, agonism by IgG C1 results in approximately half the maximal activation at saturation compared to cross‐linked tetramer clusters (Figures 2g and 8f). Similar to cross‐linked tetramers, the bouquet octamer cluster could potentially polymerize as well to create a similar functional dependence on cluster size (Figure S7D,E). We therefore concluded that proximal kinases are necessary and adequate for canonical EphA2 signaling initiation, but the active state of EphA2 could be created by any number of synthetically ordered cluster assemblies where the stochiometry of activated kinase is associated with cluster geometry and extent of EphA2 polymerization.

### 2.9 Discussion

Utilizing synthetic Abs targeting distinct epitopes of the EphA2 receptor, we developed a toolkit to antagonize and agonize receptor activation to different maxima and provided mechanistic insights into each of these processes using structural models. We deduced two distinct cluster configurations capable of agonizing the receptor. Each structurally characterized synthetic cluster relies on the same natural self‐association interfaces — LL, LL′, and CC — as Efn‐mediated clusters to recruit EphA2 protomers into signal‐competent assemblies. Modeling of the minimal signaling‐competent clusters revealed a proximity problem, whereby dimerized EphA2 is unable to recruit intracellular kinases in a manner similar to other RTK dimers. We propose that, unlike the triggering mechanism of other RTKs, EphA2 is not activated by Efn‐mediated receptor dimerization but instead relies on Efn‐mediated tetramerization to recruit a minimum of four protomers (register 1) into an active signaling complex. These seeded clusters can then further polymerize into more efficient higher ordered clusters. Our proposed EphA2 triggering model is distinct from dimer models in that EphA2 kinases are not equivalent in pTyr status within recruited clusters. Instead, pTyr modification of EphA2 occurs in the core of the EphA2 clusters where proximity of TMs and kinases can be achieved while kinases at the edges of clusters remain unmodified. We propose a structural EphA2 signaling mechanism tuned by cluster size. A key feature of this mechanism is the role of condensations to enhance pTyr efficiency, consistent with well‐established functional observations (Chen et al., 2021; Zapata‐Mercado et al., 2022). While our new model is supported by structure/function and cellular signaling evidence to date, we encourage additional validation of these principles in the context of cellular systems. Our synthetic Abs similarly demonstrate how EphA2 activity can be tuned by cluster type, where the quantity of activation observed in cells is dependent on the efficiency of the induced cluster to recruit the appropriate kinase geometry to initiate EphA2 signaling. Using this new model of activation, we can rationally engineer cluster‐inducing drugs with differential performances (Figure S7E).

Receptor recruitment by IgGs targeting three distinct epitopes created three distinct clusters that provided three distinct maximal activations (Figure 2f), demonstrating a practical method of tuning EphA2 pTyr signaling in cells and potentially *in vivo*. These distinctions in the agonistic mechanism may have significant implications for therapeutic applications. Notably, persistence of EphA2 signaling clusters upon internalization in turn regulates metastasis potential in some tumor models (Ravasio et al., 2020). In the context of EphA2 ADCs, it is therefore critical to optimally deliver conjugated payloads while minimizing tumor metastasis and limiting non‐canonical signaling. Our collection of agonists enables selective delivery of agents with distinct cluster profiles, distinct stoichiometry of recruited kinases, and distinct signaling potential for developing ADCs to evaluate *in vivo*.

Our monovalent Fab data provide evidence of two distinct inhibitory sites on EphA2, each of which overlaps with one of two binding sites for Efn. The LBD epitope has been well defined as an inhibitory epitope, being previously targeted by competitive peptide inhibitors and Abs (Goldgur et al., 2014; Gomez‐Soler et al., 2019). Similar to our observations, monovalency was a necessity for complete inhibition. Interestingly, the previously described Fab 1C1 does not act as an inhibitor, but rather, as a lower‐affinity agonist (Peng et al., 2011). This unique feature of Fab 1C1 is likely owing to V_L_‐V_L_ dimerization observed in the crystal lattice (Figure 6a) (PDB entry 3SKJ) (Peng et al., 2011). Interestingly, we observed enhancement of Efn activation in the presence of our Fab C1 (Figure 2e), which also appears to self‐associate upon EphA2 engagement. Amplification of Efn signaling by an anti‐EphA3 Ab (IIIA4) has also been reported (Vearing et al., 2005) and may be a common feature for reagents that self‐associate to induce EphA2 dimers, which in turn enhances the recruitment of tetramers by Efn. These considerations highlight the importance of Ab modality self‐association to Eph clustering and activation.

Similar to the antagonistic effect of Ab F1, FN2 has been identified as the binding site for the partial inhibitor IgG DS‐8895a, which was safely applied in a clinical trial but has not progressed further than phase I (Hasegawa et al., 2016; Shitara et al., 2019). Our experiments suggest that a range of forward signaling responses can be induced by this epitope through engineered valency (Figure S2G–I). The weak agonist activity observed for IgG F1 (Figure 2g) likely implies that bivalent molecules targeting FN2 epitopes act as weak partial agonists (Figure S2G). It still remains unclear how partial agonism and full antagonism are achieved by engaging the same site (Figures 2i and S2H,I). We presume a third form of signaling cluster is achieved with DFF F1 (Figure S7E) but further investigation of this multi‐faceted epitope of EphA2 is required.

Much like observations made with other RTKs (Merchant et al., 2013; Neijssen et al., 2021), we observed optimal inhibition using either monovalent Fab L1 at Efn Site 1 to competitively block ligand binding, or Fab F1 at Efn Site 2 to constrain the conformational freedom of FN2 and exclude proximal kinase engagement. Importantly, this is not a competitive action for closed forward signaling complexes, but rather, a non‐competitive means by which clustering can be uncoupled from kinase recruitment. However, in the context of open anti‐parallel signaling zippers where both Efn engagement sites are necessary, the response to Fab and IgG F1 is still undetermined. We anticipate that F1 Fabs will interfere with zipper formation in cellular synapses. Our study also suggests that Fab F1 does not exclude the LL/CC and LL interfaces from having antagonist activity, but instead, restricts the conformations of the receptor to exclude those that allow for TM dimerization and subsequent kinase recruitment. Non‐competitive uncoupling of EphA2 signaling by Fab F1 represents a means for EphA2 forward signaling inhibition without simultaneous inhibition of Efn reverse signaling. A similar mechanism of action may occur when cis‐engaging Efn partially inhibits Eph forward signaling (Carvalho et al., 2006) or when cis‐engaging EphA2 (head‐to‐tail) terminates signaling clusters at the auxiliary zone (Shi et al., 2023). These principles suggest a simple monovalent design strategy for inhibitory IgGs with unique mechanisms for clinical evaluation. Thus, the synthetic Abs generated here will allow further exploration of the therapeutic potential of inhibitors targeting EphA2 in cancer and infectious disease.

## 3 METHODS

### 3.1 Selection of anti‐Eph Abs

Recombinant human EphA2 fragment proteins were obtained from the Structural Genomics Consortium (Himanen et al., 2010) or were acquired commercially (R&D Systems, 3035‐A2‐100). Proteins purified from SF9 conditioned media were isolated by Ni‐NTA chromatography and size exclusion chromatography using a Superdex 200 16/60 column. Fragments were verified to be >95% pure by sodium dodecyl sulfate‐polyacrylamide gel electrophoresis (SDS‐PAGE). Fabs were selected from Fab‐phage library F using a domain exclusion method and standard methods, as described previously (Adams et al., 2014; Persson et al., 2013). Briefly, phage selections were performed using naïve library F on an EphA2 ECD domain‐truncation series (Himanen et al., 2010) as well as EphA2 ECD (R&D). Round 1 selections on truncated EphA2 were performed in a stepwise manner, whereby the naïve library was added to the LBD construct first, then the non‐binding phage were transferred to incrementally larger EphA2 fragments (Adams et al., 2014). The Eph profiler phage selections were performed using naïve library F on Eph ECDs (A1‐A8, A10, B1‐B4, and B6) (R&D) using Fc to preclear the library and ECD to positively select phage‐Fabs over four rounds of phage selections. Individual clones were identified by Sanger sequencing of the Fab V_L_ and V_H_ sequences and subcloned into a Fab expression vector for periplasmic expression.

### 3.2 Antibody expression and purification

Fab proteins used in Ab characterization assays were produced in *Escherichia coli* BL21 and purified by Protein A chromatography, as described previously (Gallo et al., 2020). IgG proteins were produced in mammalian cells and purified as described previously (Enderle et al., 2021). Fabs used for crystallization of EphA2 were produced in mammalian cells as follows: genes encoding the heavy and light chains of Fabs L1, C1, and F1 were cloned into separate vectors optimized for mammalian expression. In addition, crystallization‐enhancing substitutions (Bailey et al., 2018; Bruce et al., 2023; Lieu et al., 2020) were incorporated into the Fab framework as follows: Fab L1 (S1:Crystal‐Kappa:Elbow, and S1:Crystal‐Kappa, for the 4.60 and 2.60 Å structures, respectively); Fab C1 (S1:Crystal‐Kappa:Elbow, and S1:Crystal‐Kappa, for the 1.85 and 2.99 Å structures, respectively); and Fab F1 (Crystal‐Kappa). After growing Expi293™ cells (ThermoFisher) to a density of 2.6 × 10^6^ cell/mL in Expi293 media (Gibco), co‐transfection was carried out with the appropriate Fab heavy and light chain expression vector mixture using FectoPRO® DNA transfection kit (Polyplus transfection) according to the manufacturer's instructions. Cell growth and Fab expression proceeded for 5–6 days under the following conditions: 37°C, 8% CO_2_, 80% humidity with shaking at 125 rpm. After expression, cells were pelleted by centrifugation and the supernatant was supplemented with phosphate‐buffered saline (PBS). Fab proteins were captured on Protein A Agarose beads (Pierce) through incubation at 4°C, 125 rpm for 2 h and eluted with Pierce™ IgG Elution Buffer, followed by buffer exchange into 20 mM 4‐(2‐hydroxyethyl)‐1‐piperazineethanesulfonic acid (HEPES) pH 7.5, 100 mM NaCl using 10 kDa molecular weight concentrators (ThermoFisher Scientific #88528). Both denaturing and native polyacrylamide gel electrophoresis were used to confirm Fab and antigen purity and homogeneity. All proteins were clarified by centrifugation (13 krpm at 4°C for 30 min) prior to Fab:antigen complex preparation for crystallization screens.

### 3.3 Surface plasmon resonance

SPR measurements were performed at 25°C using a ProteOn XPR36 instrument (Bio‐Rad). EphA2 was immobilized by amine coupling to glycol/carboxyl (GLC) sensor chip surfaces. Fabs were diluted to 100 nM, and three‐fold dilutions were made with PBS buffer and injected for 1200 s at a flow rate of 50 μL/min. Dissociation in PBS buffer was monitored for up to 1200 s, and surfaces were regenerated by injection of 10 mM glycine, pH 1.5. For binding kinetics, sensorgrams were fitted to a 1:1 Langmuir model using ProteOn Manager Software (Bio‐Rad). Values for *k*_a_, *k*_d_, and *K*_D_ were calculated by taking the average of values determined by locally fitting each binding curve of a data set with globally fitted Rmax to the Langmuir binding model.

### 3.4 Biolayer interferometry

IgG specificity kinetics of binding to the EphA2 ECD were determined by BLI with an Octet HTX instrument (ForteBio) at 1000 rpm and 25°C. IgGs were captured on anti‐human biosensors from a 1 mg/mL solution in PBS, and unoccupied sites were quenched with 50 mg/mL Fc. Receptor ECDs were diluted with assay buffer (PBS, 1% bovine serum albumin (BSA), 0.05% Tween 20), and 300 nM of an irrelevant Fc‐fused protein of similar size was used as a negative control. Following equilibration with assay buffer, loaded biosensors were dipped for 600 s into wells containing three‐fold serial dilutions of each receptor ECD, starting at 300 nM, and subsequently were transferred back into assay buffer for 600 s. Binding response data were corrected by the subtraction of response from a reference and were fitted with a 1:1 binding model using ForteBio Octet Systems software 9.0.

### 3.5 Epitope binning

Epitope binning experiments were performed using an Octet HTX instrument (ForteBio) at 25°C with shaking at 1000 rpm. EphA2‐Fc protein was immobilized on AR2G (18‐5092, ForteBio) BLI sensors. Coated sensors were transferred into 100 nM IgG in assay buffer (PBS, 1% BSA, 0.05% Tween20) for 300 s to achieve saturation of binding sites. Sensors were then transferred into 100 nM competing Ab in assay buffer for 180 s. Response at 300 s after exposure to competing Ab was measured and normalized to the binding signal on unblocked antigen‐coated sensors.

### 3.6 Flow cytometry

Cells were pelleted, washed twice in PBS, 1% BSA, and stained with 200 μM primary Fab or IgG for 1 h at 4°C. Cells were washed twice with PBS, 1% BSA, and stained with human F(ab′)2‐Alexa 488 specific secondary Ab (Jackson Immunolaboratories) along with 7‐Aminoactinomycin D for 30 min at 4°C. Cells were washed twice with PBS, 1% BSA, fixed with 4% paraformaldehyde, and analyzed on a BD FACScalibur or Beckman Coulter Cytoflex. Live singlets were gated, and the median fluorescence intensity of the population was quantified and divided by that of cells stained with secondary Ab alone.

### 3.7 EphA2 pTyr stimulation

BxPC‐3 cells in 12‐well plates were grown overnight to 70% confluency in 1% fetal bovine serum (FBS) before stimulation with artificially clustered EfnA1 (positive control) and/or tested Abs at 10 nM or 100 nM. The clustered ligand was freshly prepared by mixing EfnA1‐Fc (R&D 6417A1, 100 mg/mL) with an Ab recognizing human IgG Fc (Jackson ImmunoResearch 109‐005‐098) in a 1:1.2 μg ratio for 30 min at 4°C. After incubation with EfnA1 and/or tested Abs at 37°C, 5% CO_2_ for 15 min, cells were washed and lysed for analysis using the DuoSet Human Phospho‐EphA2 ELISA (R&D Systems DYC4056‐5). For western blots, cells were lysed on ice in radioimmunoprecipitation assay (RIPA) buffer (Cell Signaling #9806) and equal amounts of total protein were resolved by SDS‐PAGE before transfer onto polyvinylidene fluoride (PVDF) membranes. Membranes were blocked in PBS, 5% BSA for 1 h at room temperature before probing for target proteins. Primary Abs used were rabbit phospho‐EphA2, Tyr588 (Cell Signaling 12677, 1:1000), rabbit GAPDH (Cell Signaling 2118, 1:20,000), rabbit phospho‐EphA2, Tyr772 (Cell Signaling, 8244S 1:1000) and rabbit phospho‐EphA2, Ser897 D9A1 (Cell Signaling 6347S 1:1000). Anti‐rabbit IgG, HRP‐linked (Cell Signaling #7074) was used as a secondary Ab at 1:30,000.

### 3.8 Size exclusion chromatography

IgG L1, C1, and F1 were each complexed with human EphA2 (R&D, Cat No: 3035‐A2) in a 1:2.5 molar excess (IgG: EphA2) on ice for 30 min in PBS buffer, pH 7.0. The tetravalent DFF F1 was complexed with human EphA2 (R&D, Cat No: 3035‐A2) in a 1:5 molar excess (Tetravalent Ab: EphA2). The complexes were subjected to size exclusion chromatography using a Superose™ 6 Increase 10/300 GL column (Cytiva, 29091596) pre‐equilibrated in PBS buffer, and elution was monitored at 216 nm on an AKTA Pure 25 FPLC system (Cytiva). A standard curve of molecular weight versus retention time was generated using a gel filtration standard (Bio‐Rad, Cat No: 151‐1901). Standards included bovine thyroglobulin (670 kDa), bovine γ‐globulin (158 kDa), chicken ovalbumin (44 kDa), horse myoglobin (17 kDa) and Vitamin B12 (1.35 kDa).

### 3.9 Crystallographic screening

Previously determined EphA2 domain boundaries (Himanen et al., 2010; Seiradake et al., 2010) were used to define favorable N‐ and C‐terminal boundaries for crystallization of EphA2 domains LBD, CRD, LBD‐CRD, and FN1 (residues 23–206, 199–326, 23–326, and 438–528, respectively). All proteins were expressed in mammalian expi293F™ (ThermoFisher) cell culture with a thrombin cleavage site at their C‐termini preceding a hexahistidine tag to facilitate downstream metal‐affinity chromatography purification and enable affinity tag cleavage using standard methods. For EphA2 CRD and LBD‐CRD protein production, cell culture was supplemented with 5 μM Kifunensine (MedChemExpress) to inhibit mannosidase I and facilitate deglycosylation after protein purification by endoH (New England Biolabs).

Protein purity and homogeneity were assessed by denaturing and native polyacrylamide gel electrophoresis. For Fab:antigen complex preparation, an optimal Fab‐antigen complex molar ratio was evaluated by titrating antigen against Fab on a polyacrylamide native gel shift assay. For the Fab‐F1:EphA2‐FN2 and Fab‐L1:EphA2‐LBD‐CRD complexes, the Fab:antigen molar ratios selected were 1:2 and 2:1, respectively. All other complexes were prepared at a 1:1.3 Fab:antigen molar ratio. The final concentration for all complexes prepared for crystallization screening was 7 mg/mL.

Crystallization was performed in sitting drops, either in 3‐lens 96‐well (SwissCI) plates using commercial 96‐well screens, including JCSG+Eco and PACT Premier (Molecular Dimensions), and SaltRX, INDEX, GRAS1, and GRAS2 (Hampton Research) set up on the Mosquito (SPT‐Labtech) robot using 0.2 μL protein/0.2 μL reservoir with a 40 μL reservoir; or crystal hits were refined further using Cryschem (Hampton Research) 24‐well sitting drops containing 1 μL protein/1 μL reservoir with a reservoir volume of 500 μL. Crystals of the EphA2 FN2/Fab^C^F1 complex were grown using a reservoir of 1.5M sodium formate, 100 mM Tris pH 8.0. Crystals grew very slowly; in fact, no crystallization occurred until after at least 1 month, after which large hexagonal plates were observed. Crystals of the EphA2 LBD‐CRD/Fab^S1C^C1 complex were observed in PACT Premier (Molecular Dimensions) condition E3, containing 20% polyethylene glycol (PEG) 3350 and 200 mM sodium iodide. Crystals of the EphA2 LBD‐CRD/Fab^S1C^L1 complex were grown in a reservoir containing 16% PEG 20 K, 100 mm HEPES pH 7.0, and 200 mM potassium thiocyanate. Crystals of the EphA2 LBD‐CRD/Fab^S1CE^L1 complex were also observed in many conditions but had poor morphology (fan‐like). However, in the presence of magnesium and calcium salts, thicker single needle crystals were observed, with the best crystals grown in a reservoir containing 16% PEG 8000, 100 mM 2‐(N‐Cyclohexylamino)ethanesulfonic acid (CHES) pH 9.0, and 200 mM magnesium sulfate. Apo Fab^S1C^L1 crystals were grown in 18% PEG 6 K, 1M lithium chloride, 100 mM sodium citrate pH 5.0, and cryoprotected in 16% PEG 6 K, 500 mM lithium chloride, 100 mM sodium citrate pH 5.0, and 25% ethylene glycol. Crystallization of the EphA2 CRD:Fab^S1CE^C1 complex and the apo Fab^S1C^C1 were described previously (Bruce et al., 2023). Following crystallization, crystals were all cryoprotected in a buffer identical to or very similar to the crystallization liquor plus 25% ethylene glycol, prior to flash‐freezing in liquid nitrogen.

### 3.10 Data collection

All data were collected at the Argonne National Laboratories beamline 24‐ID‐E. Data were either processed at the beamline using AUTOPROC (Vonrhein et al., 2011), or separately processed by Mosflm (Battye et al., 2011) or DIALS/XIA2 within CCP4 (Winter et al., 2013). Data were merged and scaled using AIMLESS (Evans & Murshudov, 2013), either within the AUTOPROC pipeline or from CCP4 (Winn et al., 2011); data resolution was based on the resolution where the overall signal to noise (*I*/*σ*) was >2 following data merging using AIMLESS (with the exception of the very low resolution EphA2 FN2/Fab^C^F1 and EphA2 LBDCRD/Fab^S1CE^L1 structures, where resolution was cut at *I*/*σ* of 1.5 and 1.3, respectively). All structures were solved by PHASER (McCoy et al., 2007) in the Phenix crystallography suite, followed by iterative cycles of refinement using phenix.refine in the Phenix suite (Zwart et al., 2008) and manual refinement of the resulting model and electron density map using the molecular graphics package COOT (Emsley & Cowtan, 2004). Translation‐libration‐screw (TLS) parameterization (Urzhumtsev et al., 2013) with group B‐factor refinement was also used. Ramachandran restraints were also used during refinement of all structures. Molecular replacement involved a number of different search models. For Fab search models for the EphA2 LBD‐CRD/Fab^S1C^C1and EphA2 LBD‐CRD/Fab^S1C^L1 datasets, Fab coordinates from PDB 8TRS were split into C_H1_/C_L_ and V_H_/V_L_ fragments; each model was separately searched during molecular replacement. For the Fab^S1C^L1 and EphA2 LBD‐CRD/Fab^S1CE^L1 complex datasets, Fab coordinates from the refinement of the EphA2 LBD‐CRD/Fab^S1C^L1 structure were split into C_H1_/CL and V_H_/V_L_ fragments for molecular replacement. However, this strategy of splitting the Fab into two pieces was not successful with the lower resolution data of the EphA2‐FN2/Fab^C^F1 as it would require eight Fab search models for ~4 Å data. Instead, coordinates from a 3.5 Å structure of Fab^S1^F1 (PDB entry 8T7F) (Bruce et al., 2023) (chains C and G, heavy and light chain together as a single search) was used. In terms of EphA2 search models, residues 25–325 of PDB 3FL7 initially represented the EphA2 LBD‐CRD in the EphA2 LBD‐CRD/Fab^S1C^C1, EphA2 LBD‐CRD/Fab^S1C^L1, and EphA2 LBD‐CRD/Fab^S1CE^L1 data. However, during refinement of the EphA2 LBD‐CRD/Fab^S1C^L1 structure, we observed that one of the LBD‐CRD molecules seemed to undergo an internal rotation relative to the LBD‐CRD search model. Thus, for molecular replacement, the PDB 3FL7 search model was split into the LBD (residues 27–201) and CRD (residues 203–308) and searched separately. For the EphA2‐FN2/Fab^C^_F1 structure, the model of FN2 was based on a SWISS‐MODELER (Biasini et al., 2014) model of residues 438–528 of EphA2.

## AUTHOR CONTRIBUTIONS

**Jarrett J. Adams:** Conceptualization; investigation; writing – original draft; methodology; visualization; formal analysis; project administration; data curation; supervision. **Heather A. Bruce:** Methodology; investigation; formal analysis; data curation. **Suryasree Subramania:** Investigation; visualization; methodology; formal analysis; data curation. **Lynda Ploder:** Investigation; methodology; formal analysis; data curation. **Julia Garcia:** Investigation; methodology; formal analysis; data curation. **Isabelle Pot:** Writing – original draft; funding acquisition; visualization; project administration. **Levi L. Blazer:** Investigation; supervision; formal analysis; project administration. **Alexander U. Singer:** Data curation; formal analysis; investigation; methodology. **Sachdev S. Sidhu:** Conceptualization; funding acquisition; writing – original draft; visualization; formal analysis; data curation; supervision.

## CONFLICT OF INTEREST STATEMENT

The authors declare no conflicting interests.

## Supporting information


**Data S1.** Supporting Information.

## Data Availability

All sequences, structure factors, and models have been submitted to the rcsb protein data bank (www.rcsb.org) with identifiers 8T9B, 8TS5, 8TRS, 8TRT, 8TRV, 8TV2, 8TV1, and 8TV5.
