# Supplementary material for: Synthetic antibodies targeting EphA2 induce diverse signaling‐competent clusters with differential activation
Source: Protein Sci. 2025 May 24;34(6):e70145. doi: 10.1002/pro.70145 (PMC12102760; doi:10.1002/pro.70145)

**Supplemental Materials**

**
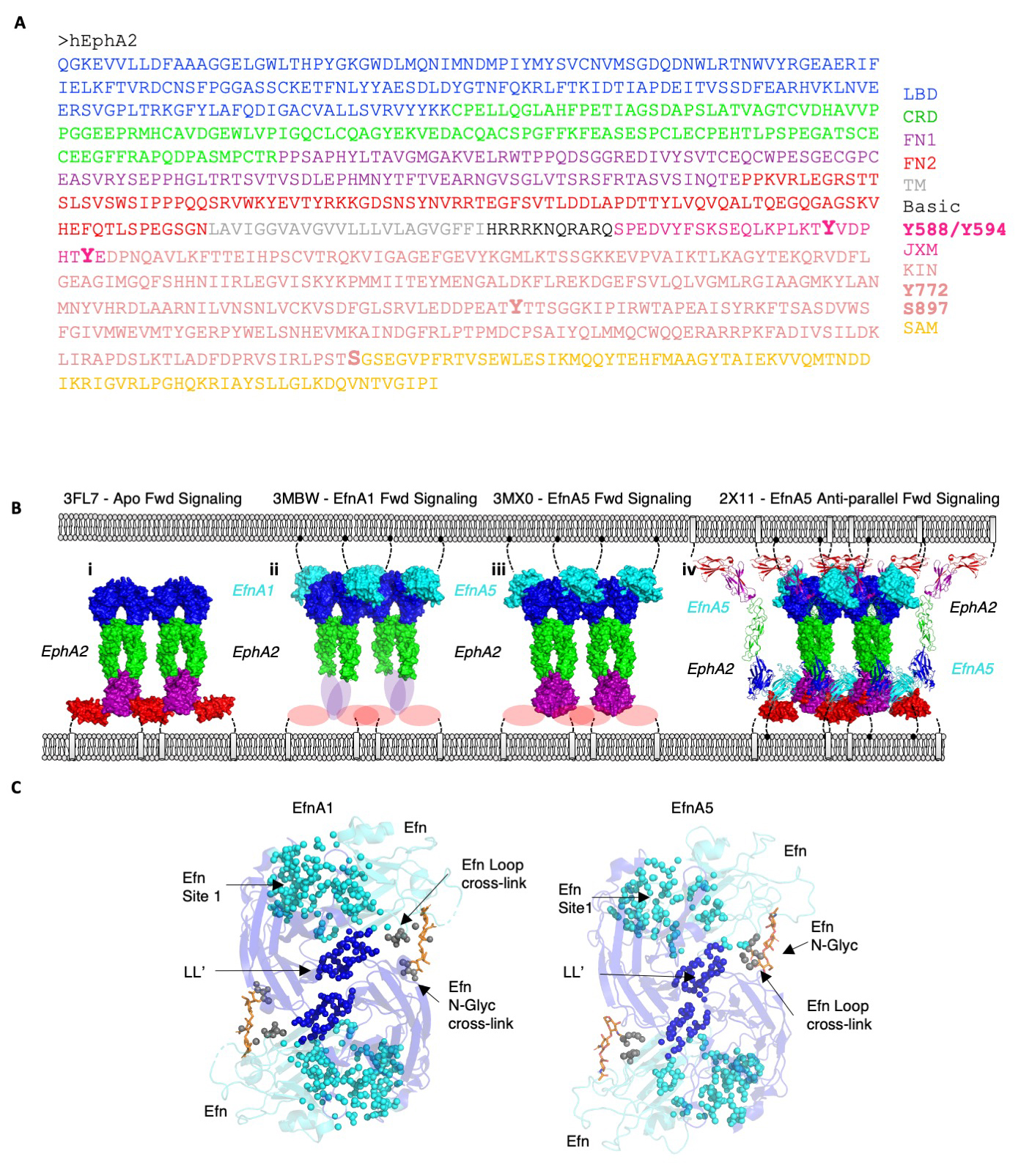
**

**Supplemental Figure S1. Conserved clustering in EphA2 complexes**

(**A**) The primary amino acid sequence of EphA2 corresponding to recombinant EphA2 is presented in FASTA format, with domains colored as indicated on the right, as follows: LBD (blue), CRD (green), FN1 (purple), FN2 (red), transmembrane (TM, grey), acidic membrane-associated region (Basic, black), tyrosine phosphorylation sites (Y^588^ and Y^594^, bold pink), juxtamembrane region (JXM, pink), kinase including tyrosine and serine phosphorylation sites (KIN, Y^772^ and S^897^, salmon), and sterile alpha motif (SAM, gold). (**B**) Conservation of tetrameric architectures observed in EphA2 clusters derived from (**i**) the apo EphA2 ECD complex (PDB entry 3FL7) and (**iv**) the EphA2-EfnA5 complex (PDB entry 2X11) compared to partial EphA2 ECD fragment complexes bound to (**ii**) EfnA1 (PDB entry 3MBW) or (**iii**) EfnA5 (PDB entry 3MX0). Domains of EphA2 including the LBD (blue), CRD (green), FN1 (purple) and FN2 (red) are illustrated, as well as EfnA5 (cyan), as tetrameric chain-link clusters observed in each lattice. Domains missing in the partial ECD complexes are approximated using colored ovals. (**C**) Ribbon diagrams of EphA2 LBD dimers mediated by EfnA1 (left, PDB entry 3MBW) or EfnA5 (right, PDB entry 3MX0) through glycan contacts to stabilize the LL’ EphA2 interface. Primary contacts ($\leq$4.5 Å) of the Efn Site 1 interface are represented as cyan spheres. Cross-linking of EphA2 by Efn loop contacts or NGlyc that cross-link the heterodimers to stabilize an LL’ interface are represented by grey spheres. LL’ dimer contacts induced by Efn cross-linking are represented by blue spheres. The EphA2 LBD is colored blue, Efn is colored cyan, glycosylation is colored orange.

**
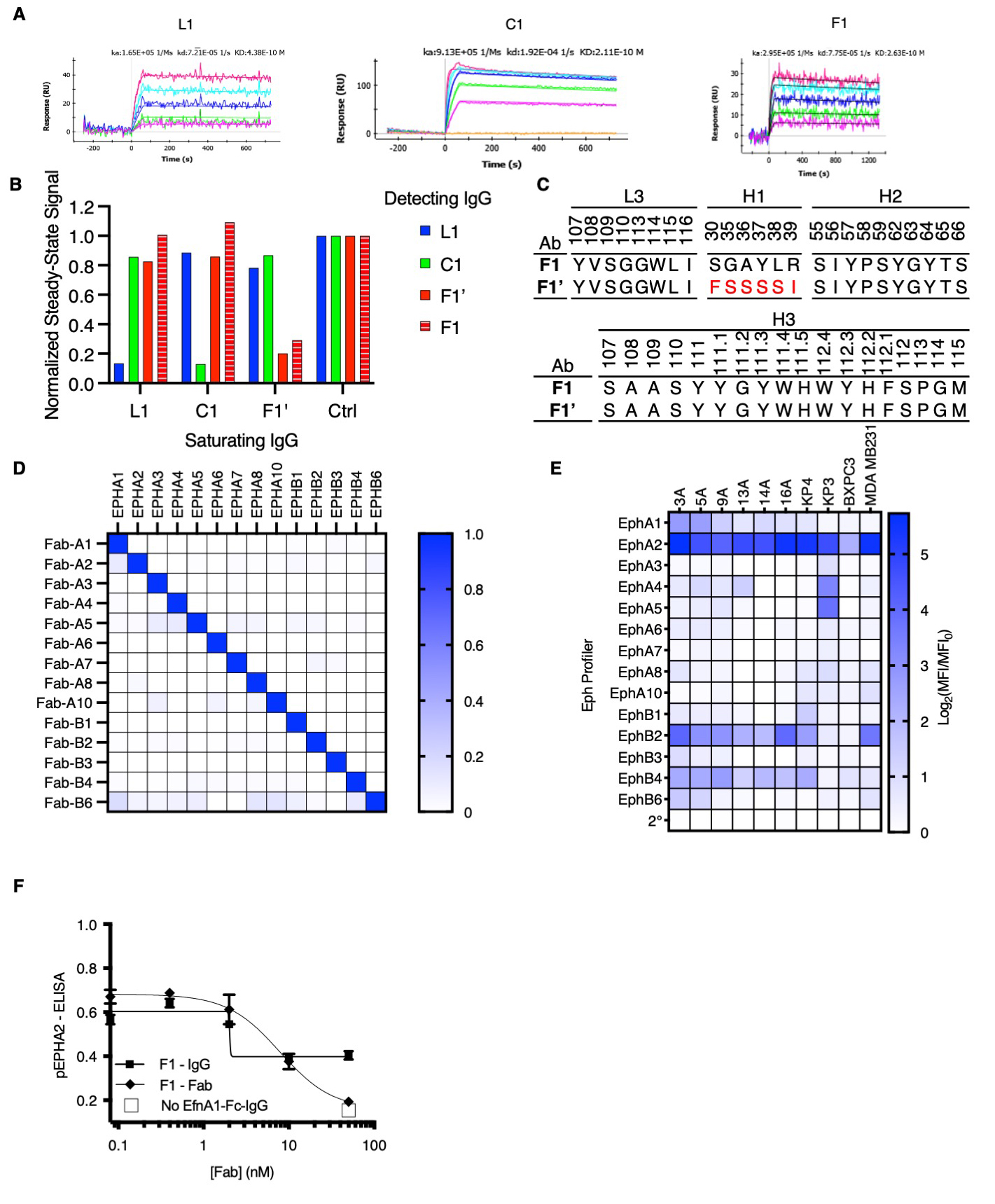

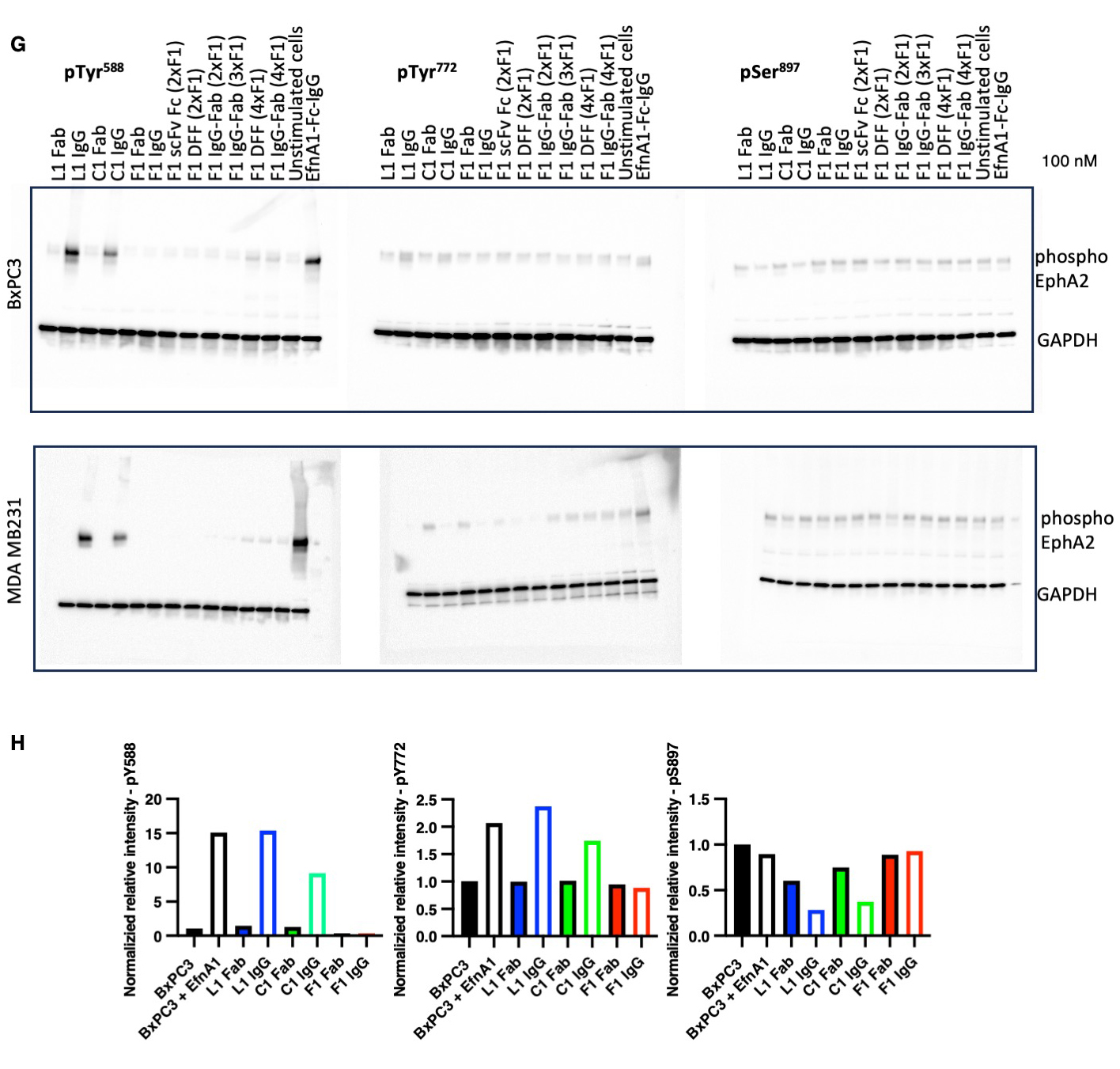

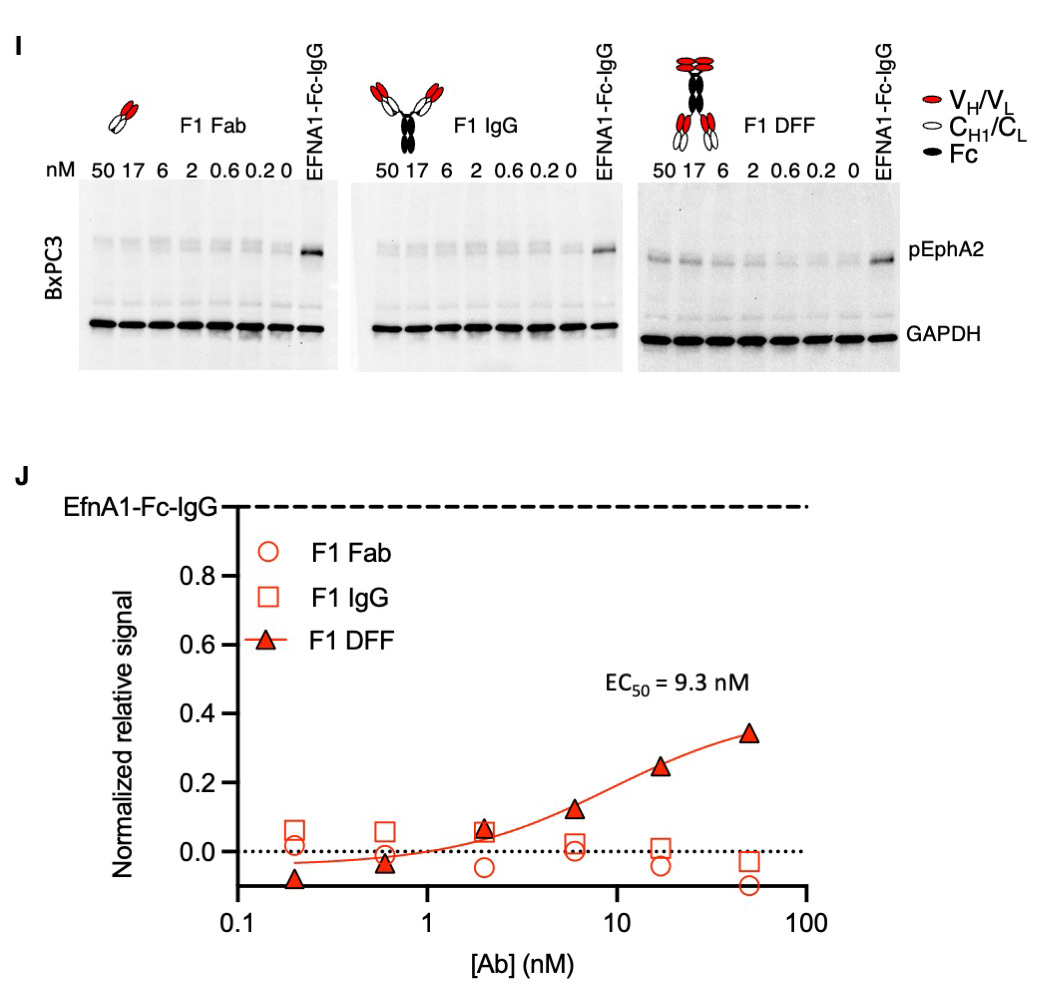
**

**Supplemental Figure S2. Affinities of Fabs for the EphA2 ECD and cellular responses.**

(**A**) SPR sensorgrams for Fabs L1 (left), C1 (center), and F1 (right) binding to two-fold serial dilutions of the EphA2 ECD (100, 50, 25, 12.5, 6.25 nM). Fabs were conjugated to anti-Fc-coated surfaces. (**B**) Epitope binning of IgGs on full length EphA2 ECD protein. Binding of IgGs (x-axis) to EphA2 ECD in the absence or presence of L1 (blue), C1 (green), F1’(red) and F1 (red stripes). The binding signal (y-axis) was normalized to the binding signal in the absence of competing ligand. Competition was determined by BLI signal at 100 nM. (**C**) CDR sequences F1 (optimized) and F1’ (parental) demonstrating the divergence in H1 sequences. (**D**) Development of an Eph Fab profiler. Receptor specificity was determined by normalized steady-state BLI interferometry for each of the immobilized Eph receptor ECDs (x-axis) binding to 100 nM of a solution-state Fab specific for one of the Eph receptors (y-axis). (**E**) Flow cytometry detection of cell-surface Eph receptors (y-axis) on pancreatic cell lines (x-axis) using the Eph Fab profiler, where fold signal over isotype control is plotted. (**F**) Effects of Fab and IgG F1 targeting FN2 (x-axis) on phosphorylation level of EphA2 in BxPC3 cells stimulated with EfnA1-Fc-IgG, assessed by quantitative ELISA detection of EphA2 pTyr^588^ (y-axis). Statistical analysis was carried out using ANOVA (*n* $\geq$ 3) in Prism Graph Pad. (**G**) Phospho-EphA2 (pEphA2) Western analysis of Ab modalities stimulating EphA2 signaling in either BxPC3 (top) or MDA MB231 (bottom). Serum starved cells were incubated with 100 nM of each modality for 15 mins. Each blot was tested for signal with secondary Abs specific for pTyr^588^, pTyr^772^, and pS^897^ signaling in response to Abs or EfnA1-Fc-IgG stimulation. Each lane was simultaneously probed for GAPDH (loading control). (**H**) Densitometry of L1, C1 and F1 IgG and Fab pEphA2 responses in BxPC3. (**I**) Titration (50-0.2 nM) of F1 modalities with increasing valency including Fab (left), IgG (middle) and DFF (right) on BxPC3 cells and detection of EphA2 pTyr^588^ and GAPDH (load control) by Western blot. (**J**) Densitometry of pEphA2 responses to titrations of F1 modalities on BxPC3.

**
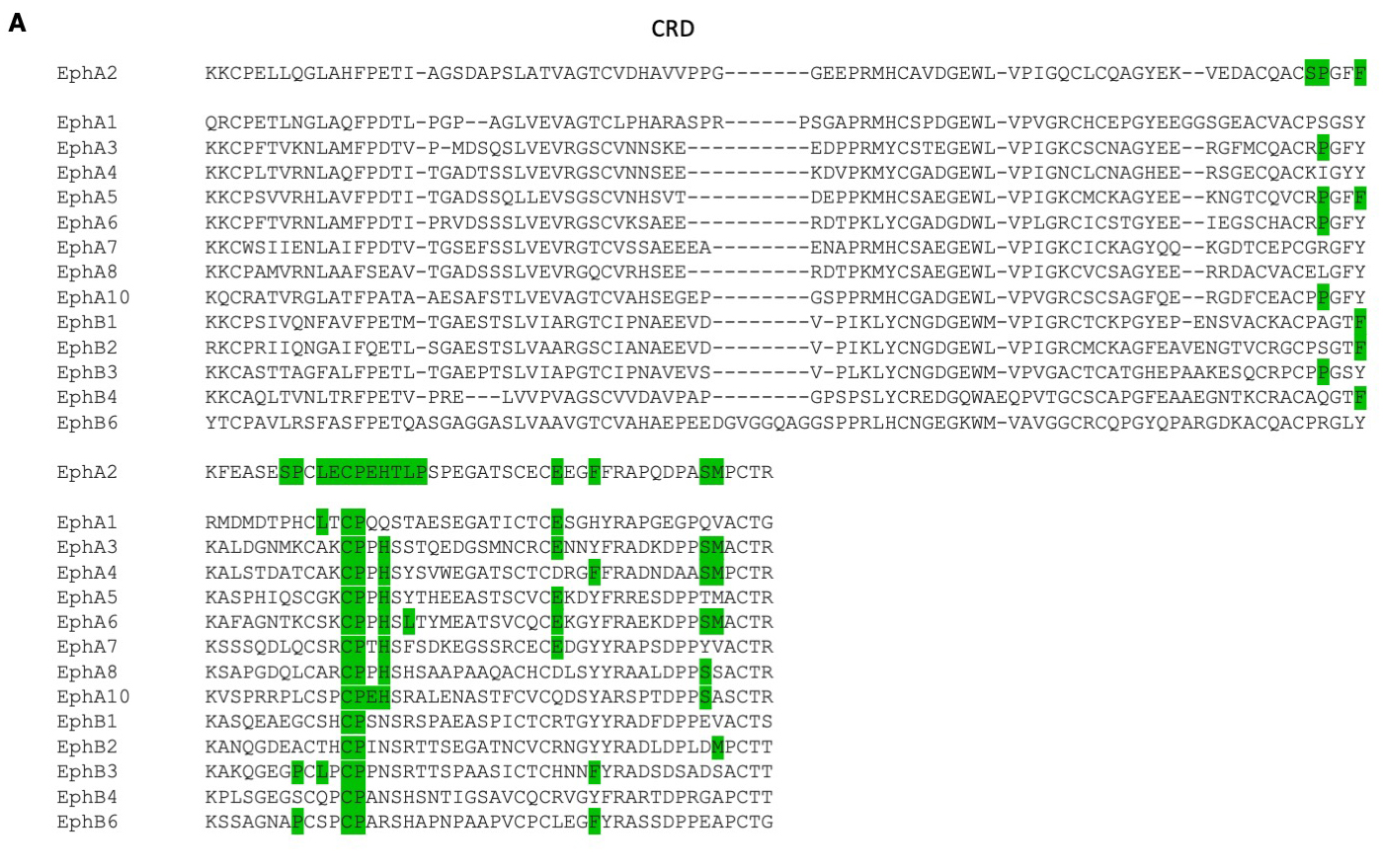
**

**
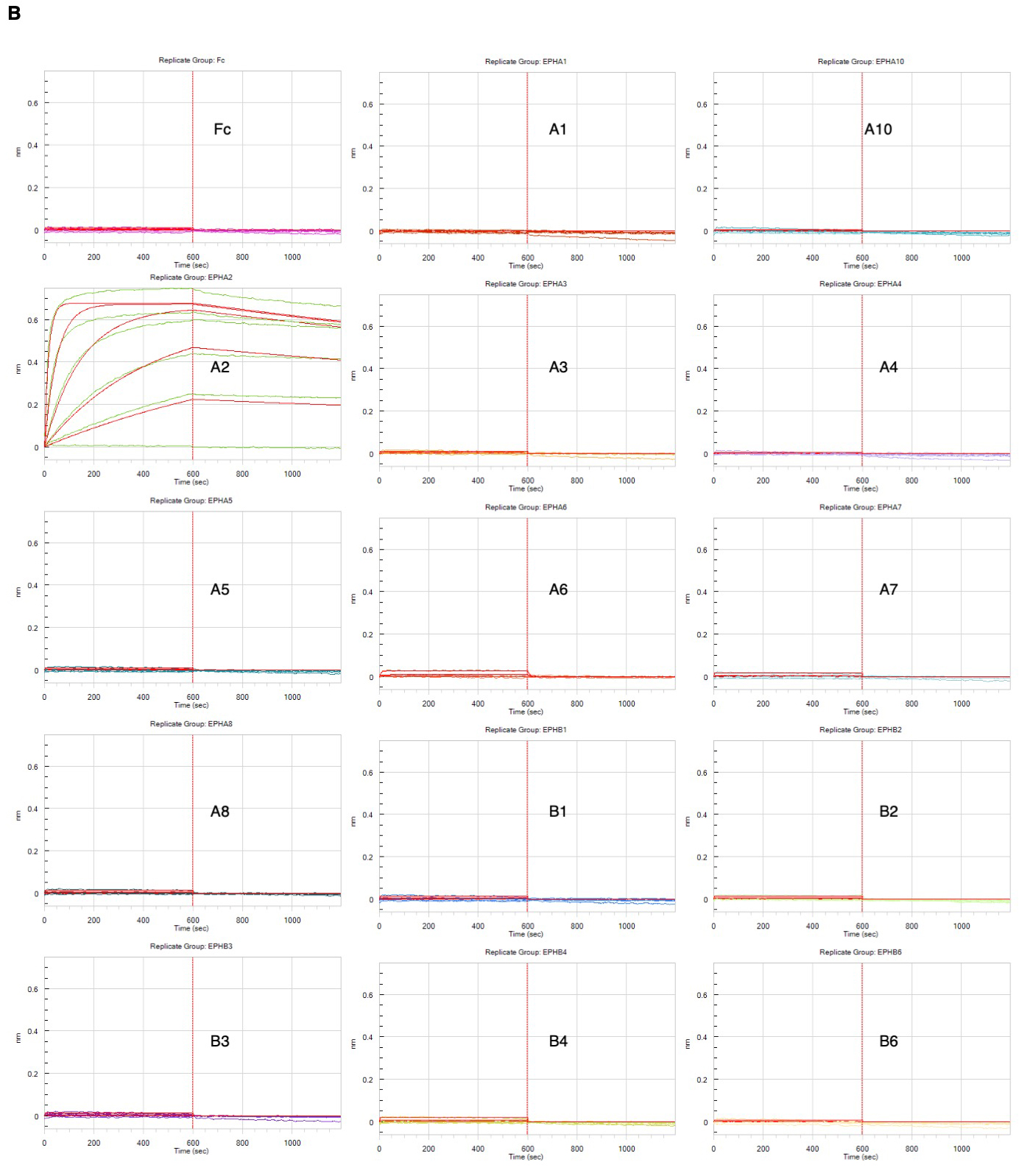
**

**
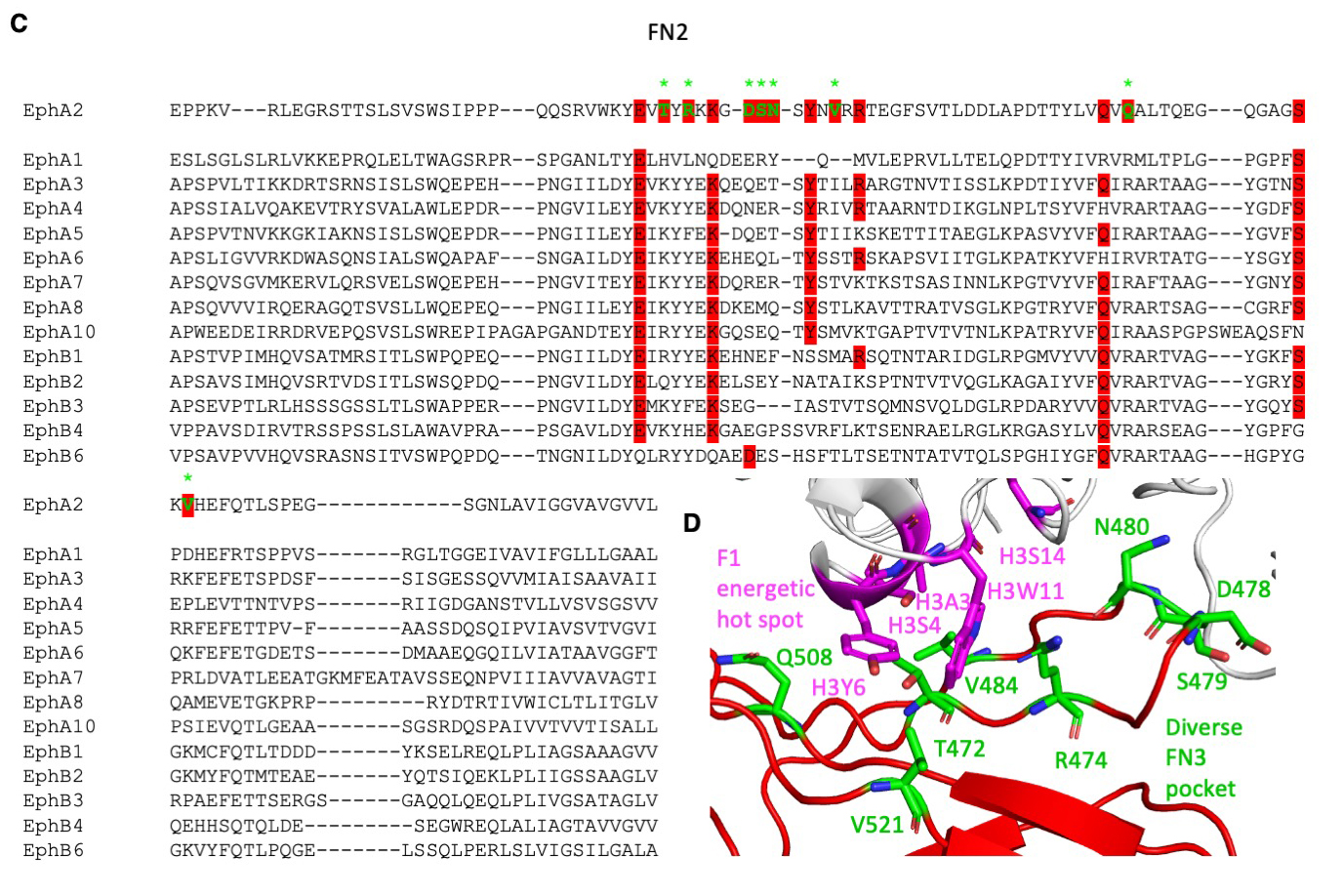
**

**
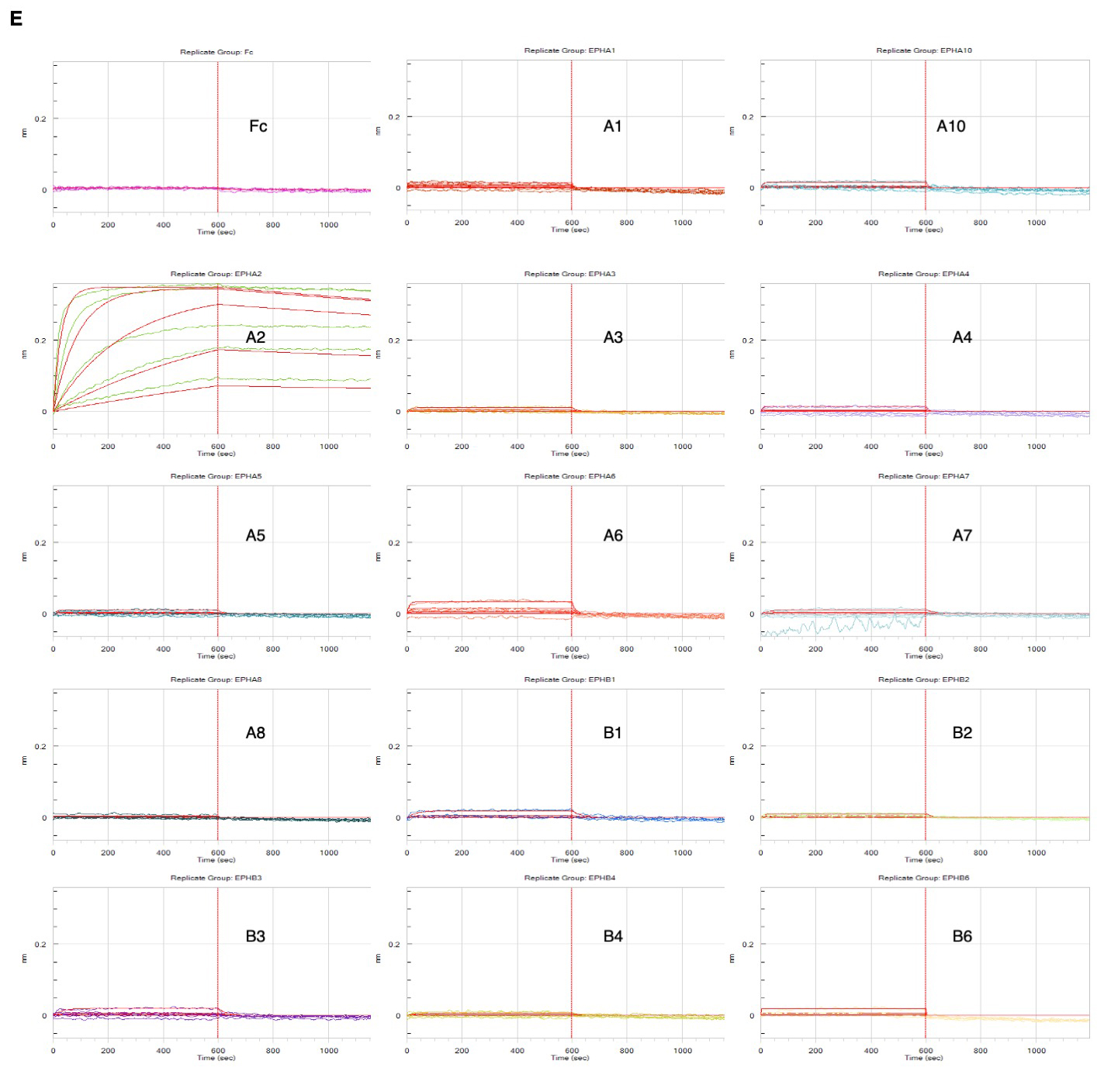
**

**
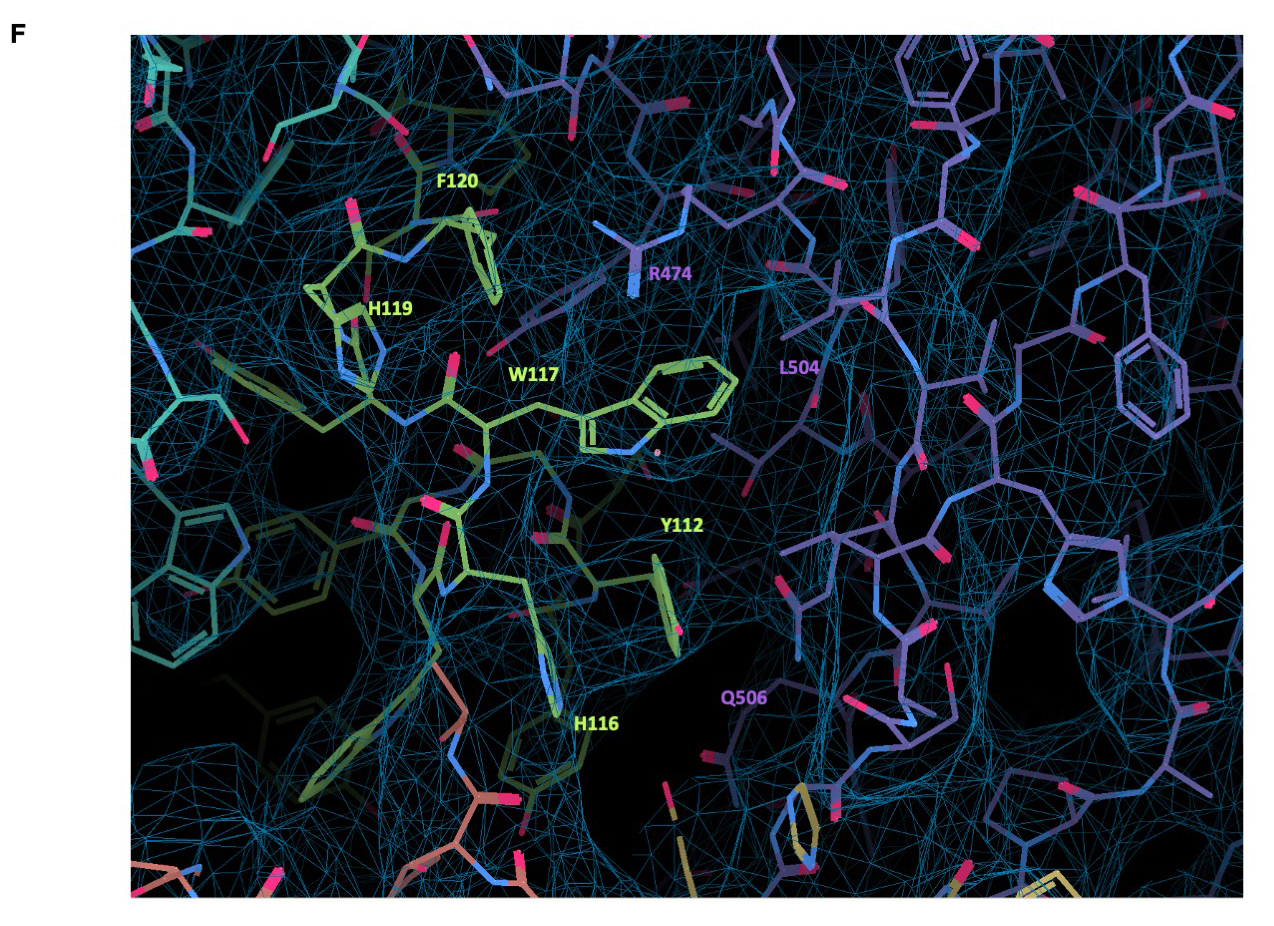
**

**Supplemental Figure S3. Specificity of IgGs C1 and F1 across the Eph receptor family**

(**A**) Sequence alignment of CRDs of the Eph family. Residues of the EphA2 epitope targeted by Fab C1 are highlighted green, as are corresponding residues that are conserved in other Eph family members. (**B**) BLI sensorgrams of titrations of EphA1-Fc, EphA2-Fc, EphA3-Fc, EphA4-Fc, EphA5-Fc, EphA6-Fc, EphA7-Fc, EphA8-Fc, EphA10-Fc, EphB1-Fc, EphB2-Fc, EphB3-Fc, EphB4-Fc, EphB6-Fc and control Fc (300-3.7 nM) binding to immobilized IgG C1. (**C**) Primary sequence alignment of FN2 domains of the Eph family. Residues of the EphA2 epitope targeted by Fab F1 are highlighted red, as are corresponding residues that are conserved in other Eph family members. Amino acids poorly conserved across FN2 isoforms are shown in green and labelled with an asterix. (**D**) Interface of the CDR-H3 hot spot on the diversity pocket of Eph isoforms. Residues of F1 (white) that contribute significantly to the energetic of binding (magenta, sticks) engage a region of EphA2 FN2 (red) that is poorly conserved across human Eph isoforms (green, sticks). (**E**) BLI sensorgrams of titration of EphA1-Fc, EphA2-Fc, EphA3-Fc, EphA4-Fc, EphA5-Fc, EphA6-Fc, EphA7-Fc, EphA8-Fc, EphA10-Fc, EphB1-Fc, EphB2-Fc, EphB3-Fc, EphB4-Fc, EphB6-Fc and control Fc (300-3.7 nM) binding to immobilized IgG F1. (**F**) Simple composite omit map of the EphA2 FN2/Fab F1 structure calculated according to Afonine et al. (2018)*, omitting 5% of the atoms iteratively. 2f_o_-f_c_ electron density for a region of the FN2/Fab interface is contoured at σ=1.0.

*Afonine, P.V., Poon, B.K., Read, R.J., Sobolev, O.V., Terwilliger, T.C., Urzhumtsev, A. & Adams, P.D. Real-space refinement in PHENIX for cryo-EM and crystallography. Acta Crystallogr D Struct Biol 74, 531-544 (2018).

**
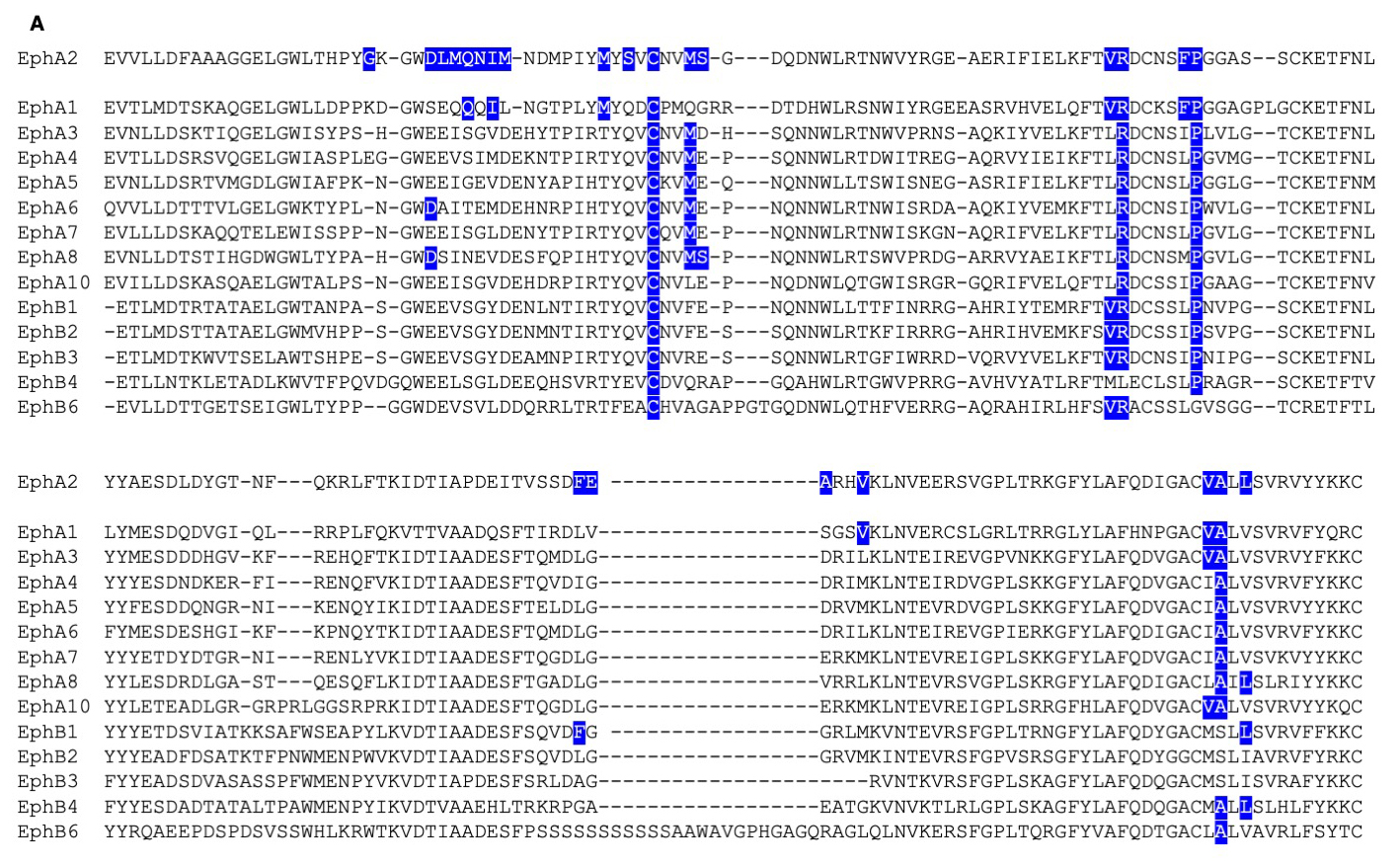
**

**
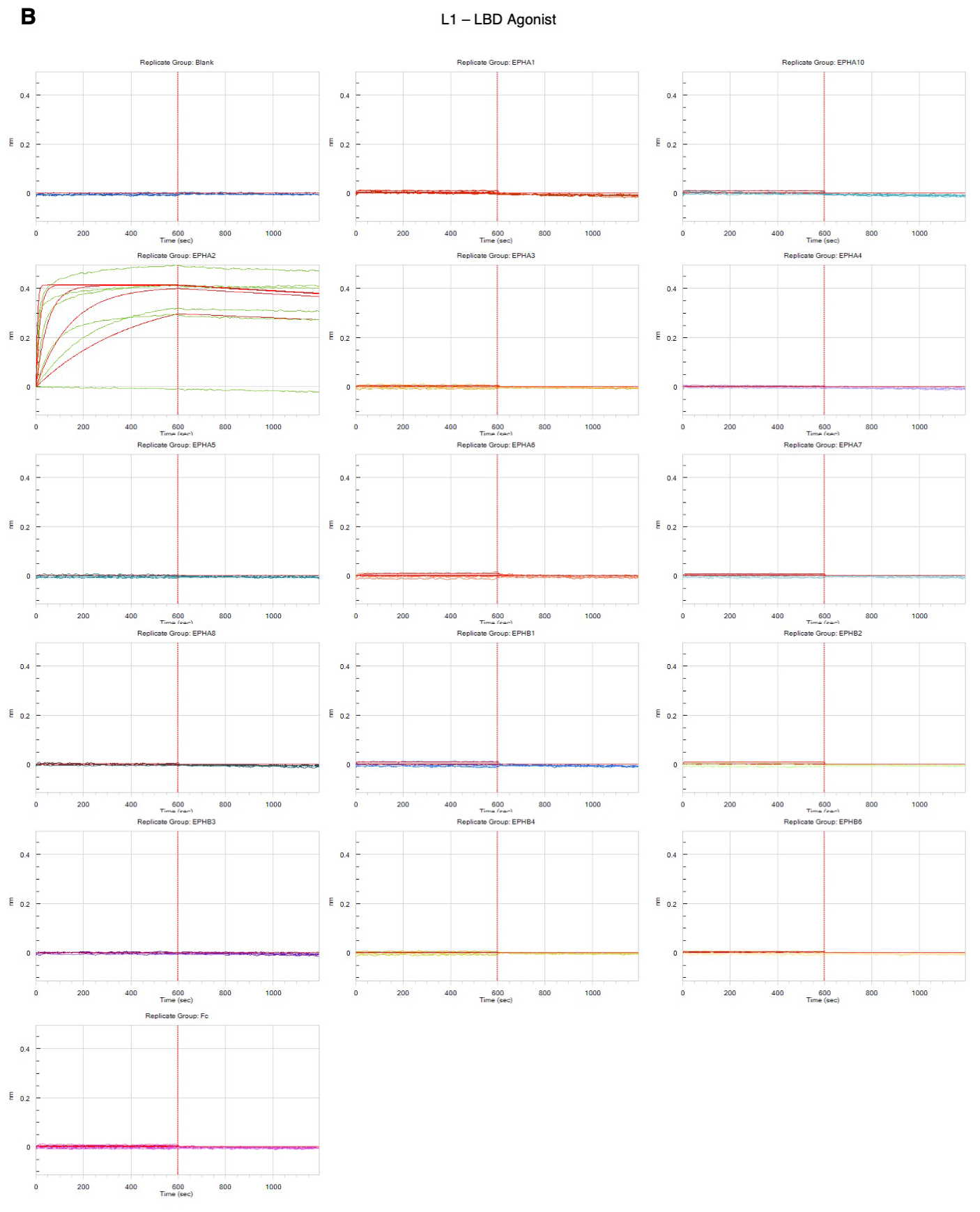
**

**
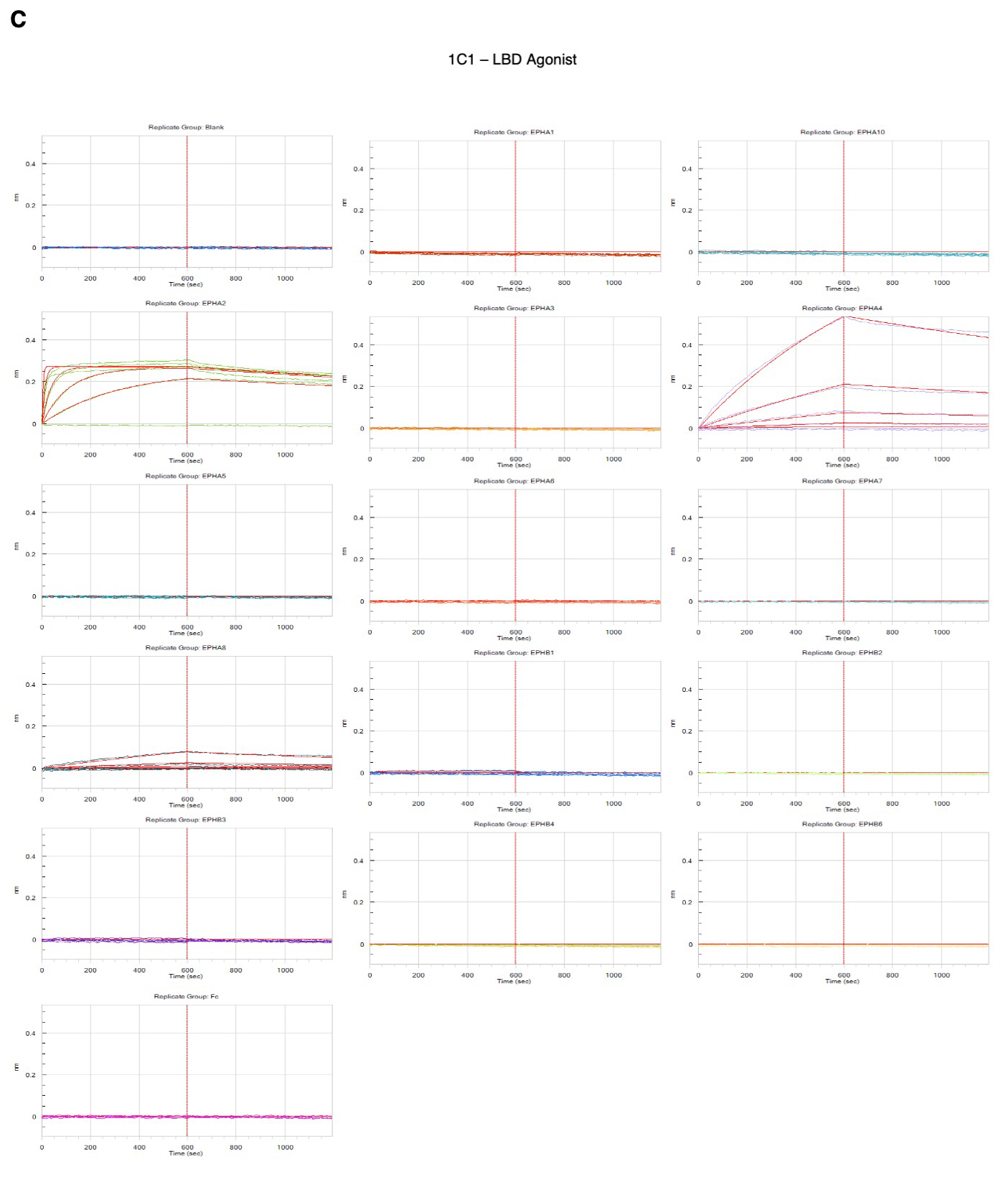
**

**Supplemental Figure S4. Specificities of IgGs L1 and 1C1 across the Eph receptor family**

**(A)** Sequence alignment of LBDs of the human Eph receptor family. The epitope residues targeted by Fab L1 are colored blue, as are corresponding residues that are conserved in other Eph family members. **(B,C)** BLI sensorgrams of titrations of EphA1-Fc, EphA2-Fc, EphA3-Fc, EphA4-Fc, EphA5-Fc, EphA6-Fc, EphA7-Fc, EphA8-Fc, EphA10-Fc, EphB1-Fc, EphB2-Fc, EphB3-Fc, EphB4-Fc, EphB6-Fc, and control Fc (300-3.7 nM) binding to immobilized IgG L1 (**B**) or 1C1 (**C**).


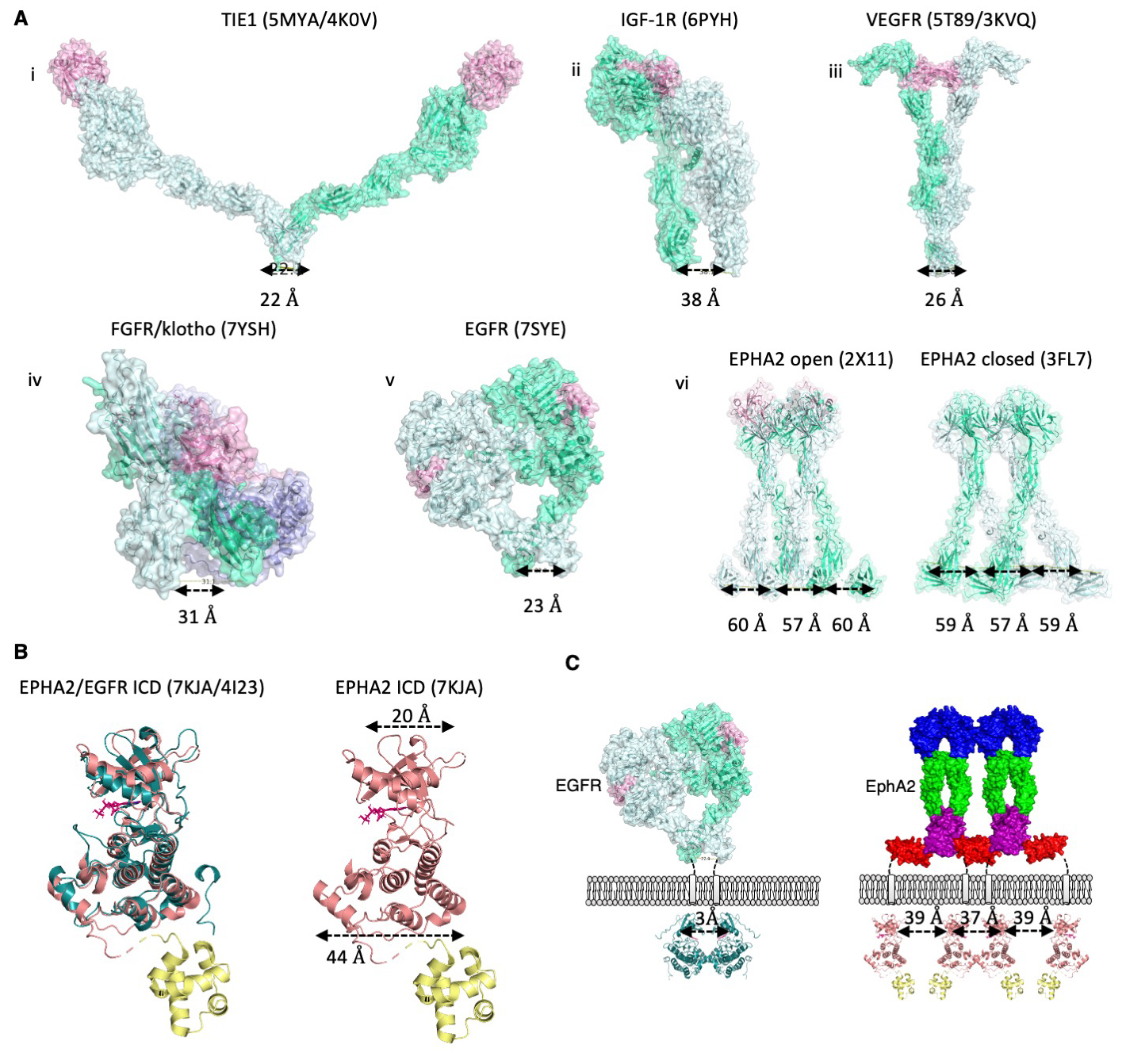


**Supplemental Figure S5. Proximity of signaling competent RTK dimers and alternative pTyr and pSer EphA2 in BxPC3**

(**A**) Surface renderings of signaling competent RTK dimers for (i) Tie1 (superposition of PDB entries 5MYA and 4K0V), (ii) IGF-1R (PDB entry 6PYH), (iii) VEGFR (superposition of PDB entries 5T89 and 3KVQ), (iv) FGFR/klotho (PDB entry 7YSH), (v) EGFR (PDB entry 7SYE), (vi) open and closed EphA2 tetramers. RTK receptors are illustrated at pale cyan and teal cyan and ligands are illustrated at magenta transparent surfaces. TM distances are estimated by the distances of the the most membrane proximal residues modeled in density. (**B**) Superpostion of the EGFR kinase ICD (dark teal) and EphA2 kinase ICD (salmon) (left). (**C**) In silico modelling of kinase domain recruitment of EGFR (left) compared cross-linked tetramers (right).

**
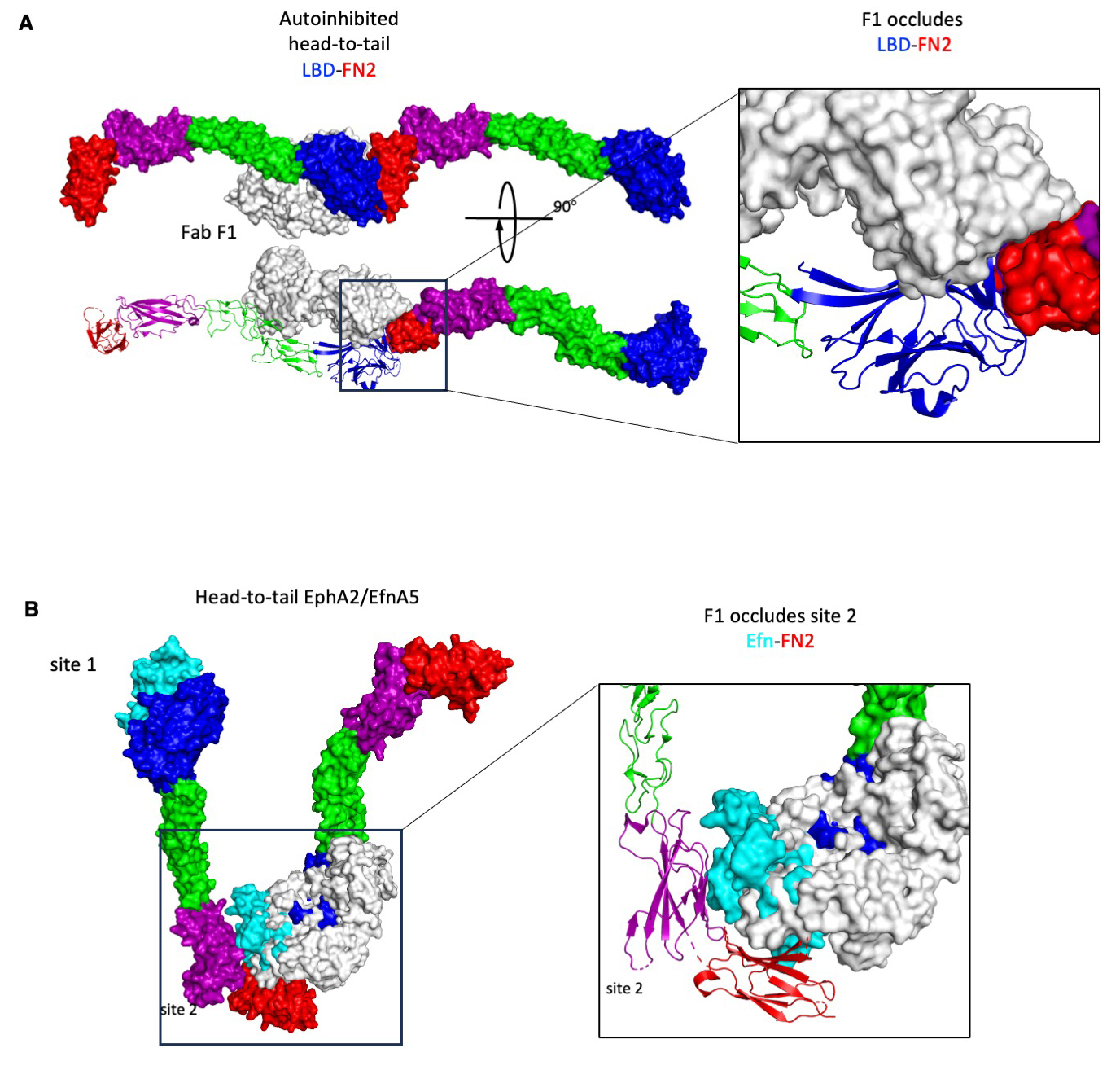
**

**Supplemental Figure S6. Alternative FN2 interfaces**

(**A**) Surface rendering of the F1 Fab superimposed onto the autoinhibitory dimer complex of EphA2 where the LBD associates with FN2. Inset is close up image of the steric clashing between LBD and Fab F1. **(B)** Surface rendering of the F1 Fab superimposed onto the complex of EphA2 and EfnA5 at site 2. Inset is close up image of the steric clashing between EfnA5 and Fab F1.

**
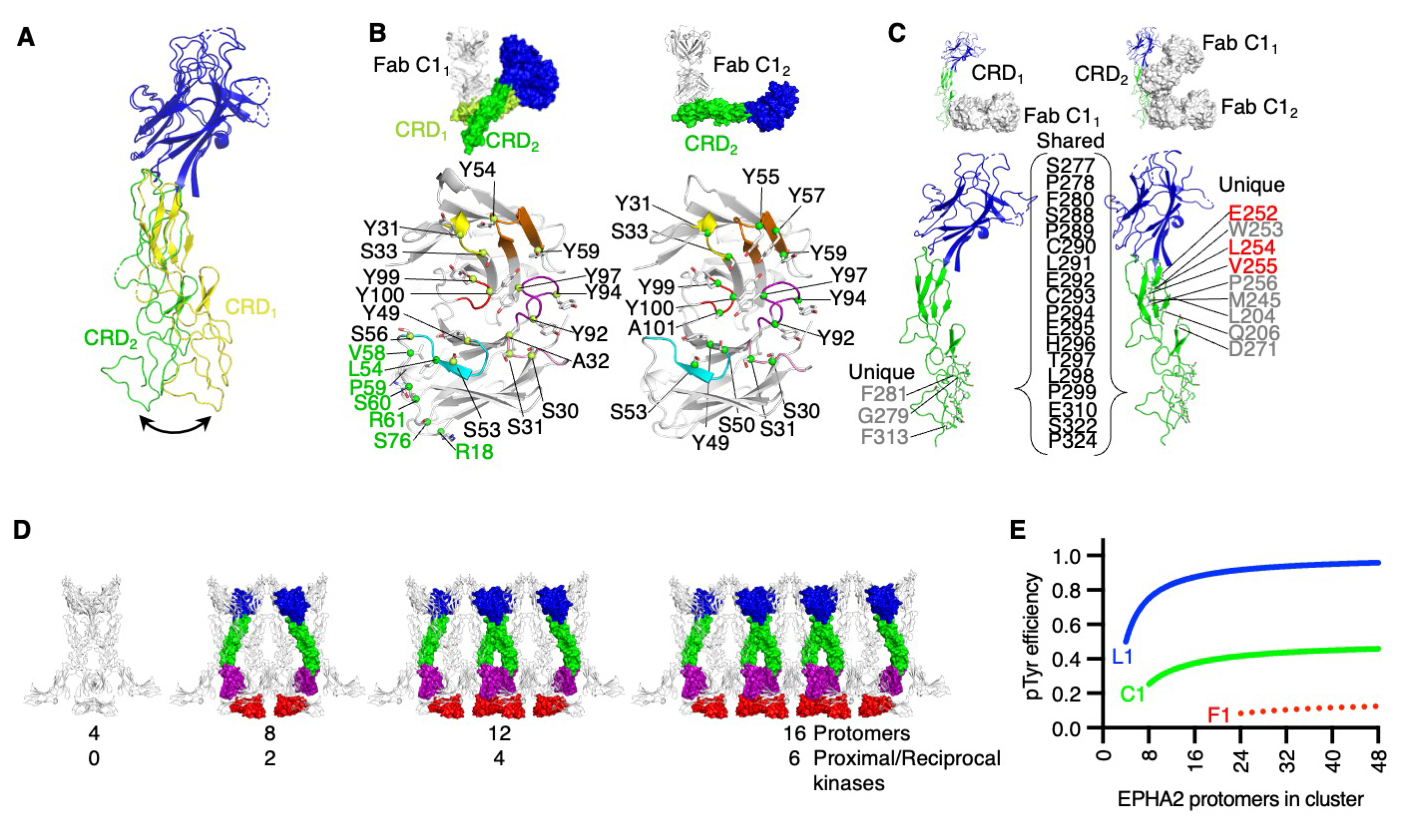
**

**Supplemental Figure S7. Distinct interfaces of the partial agonist clusters**

(**A**) Overlay of the unique conformations LBD-CRD_1_ (blue-yellow) and LBD-CRD_2_ (blue-green). (**B**) Unique paratopes of Fabs C1_1_ (left) and C1_2_ (right). Open book renderings of the paratope contact residues (<4.5 Å) are shown where CDR loops are colored as follows: CDR-H1 (yellow), CDR-H2 (orange), CDR-H3 (red), CDR-L1 (pink), CDR-L2 (cyan), and CDR-L3 (purple). Contact residues are annotated by colored Cα spheres and shown as sticks. Contact residue interactions with CRD_1_ (limon spheres) at the EGF domain and CRD_2_ (green spheres) at the sushi domain are differentially labelled black and green, respectively. Inset (above) are the ribbons-on-surfaces for the Fabs and bound EphA2 protomers, respectively. (**C**) Unique epitopes of CRD_1_ (left) and CRD_2_ (right). Contact residues are annotated by colored Cα spheres and shown as sticks. Unique contact residues and shared contact residues are labelled grey or black, respectively. Contact residues that occlude the CC interface are labelled red. Insets (above) are the ribbons-on-surfaces for the LBD-CRD and bound Fab protomers, respectively. (**D**) Profile surface renderings of bouquet clusters of increasing size (left to right) with stoichiometry. Distally recruited protomers are colored white. (**E**) A comparison of predictive models of pTyr efficiency across a range of chain-linked, bouquet and bunch clusters of EphA2.


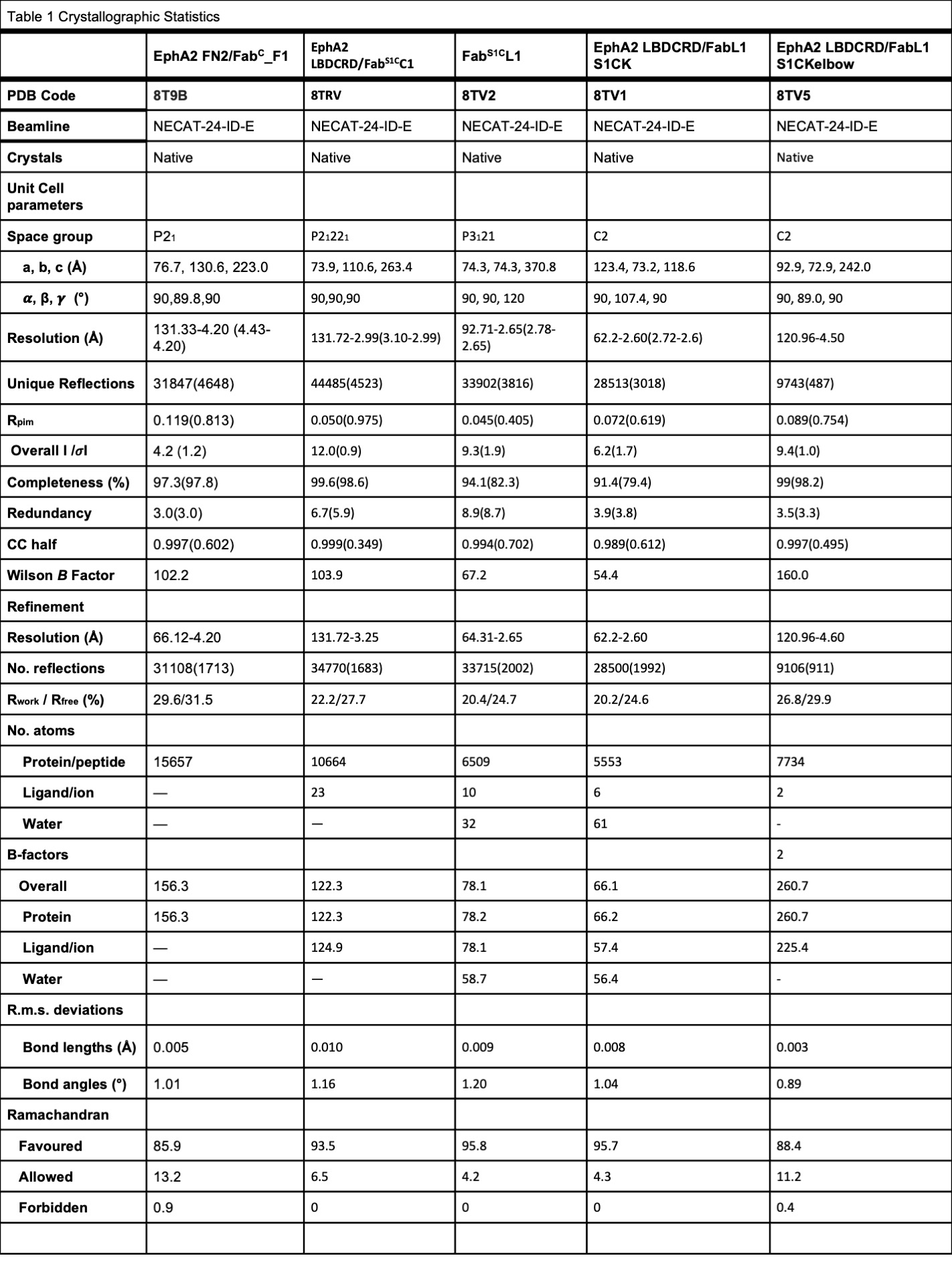

Supplement: Supplementary file 1 — Data S1. Supporting Information. [file PRO-34-e70145-s001.docx]
